# Quercetin Attenuates the Production of Pro-Inflammatory Cytokines in H292 Human Lung Epithelial Cells Infected with *Pseudomonas aeruginosa* by Modulating ExoS Production

**DOI:** 10.4014/jmb.2208.08034

**Published:** 2023-01-27

**Authors:** Hye In Ahn, Hyun-Jae Jang, Ok-Kyoung Kwon, Jung-Hee Kim, Jae-Hoon Oh, Seung-Ho Kim, Sei-Ryang Oh, Sang-Bae Han, Kyung-Seop Ahn, Ji-Won Park

**Affiliations:** 1 Natural Medicine Research Center, Korea Research Institute of Bioscience and Biotechnology, Cheongju 28116, Republic of Korea; 2 College of Pharmacy, Chungbuk National University, Cheongju 28160, Republic of Korea; 3 Life Science Research Center, Nine Biopharm Co., LTD. Cheongju 28161, Republic of Korea; 4 Practical Research Division, Honam National Institute of Biological Resources (HNIBR), Mokpo 58762, Republic of Korea

**Keywords:** *Pseudomonas aeruginosa* (*P. aeruginosa*), type three secretion system (T3SS), exoenzyme S (ExoS), quercetin, NF-κB

## Abstract

The type three secretion system (T3SS) is a major virulence system of *Pseudomonas aeruginosa* (*P. aeruginosa*). The effector protein Exotoxin S (ExoS) produced by *P. aeruginosa* is secreted into the host cells via the T3SS. For the purpose of an experiment on inhibitors with regard to ExoS secretion, we developed a sandwich-type enzyme-linked immunosorbent assay (ELISA) system. Quercetin was selected because it has a prominent ExoS inhibition effect and also is known to have anti-inflammatory and antioxidant effects on mammalian cells. In this study, we investigated the effects of quercetin on the expression and secretion of ExoS using ELISA and Western blot analysis methods. The results showed that the secretion of ExoS was significantly decreased by 10 and 20 μM of quercetin. Also, *popB*, *popD*, *pscF*, and *pcrV* which are composed of the T3SS needle, are reduced by quercetin at the mRNA level. We also confirmed the inhibitory effect of quercetin on cytokines (IL-6, IL-1β, and IL-18) in *P. aeruginosa*-infected H292 cells by real-time polymerase chain reaction (PCR) and ELISA. Collectively, quercetin inhibits the secretion of ExoS by reducing both ExoS production and the expression of the needle protein of T3SS. Furthermore, these results suggest that quercetin has the potential to be used as an anti-toxic treatment for the inflammatory disease caused by *P. aeruginosa* infection.

## Introduction 

*Pseudomonas aeruginosa* (*P. aeruginosa*) is a type of gram negative bacteria found commonly in the environment, such as in soil and water. *P. aeruginosa* is an opportunistic pathogen that causes secondary infections in immuno-compromised patients, including pneumonia and cystic fibrosis (CF). For CF, in more than 60% of cases, where the host immune response is compromised, *P. aeruginosa* presents itself as a dreadful threat and leads to a progressive decline in lung function [1-3].

*P. aeruginosa* has a variety of systems by which it can cause infection. Among virulence factors, the T3SS is especially relevant, being one of the most important virulence factors in *P. aeruginosa*. T3SS forms a needle complex that consists of proteins, including PscF, PcrV, and PopB/D on the bacterial membrane and that is regulated by ExsA, the transcription factor of T3SS [4]. In addition, T3SS is regulated by various environmental factors, such as contact with host cells and a low concentration of calcium. The T3SS needle complex acts as a translocator of four T3SS toxins, exoenzyme S, T, U, and Y, and these toxins play key roles in the virulence of *P. aeruginosa* [5-7]. Exoenzyme S (ExoS) shows ADP-ribosyltransferase (ADPRT) activity, and is typically found in patients with acute lung injuries who are also infected with *P. aeruginosa* among these secretory toxins [8, 9]. ExoS is known to convert pro-interleukin (IL-1β) into mature IL-1β via NLRC4 or NLRP3 and caspase-1 in mammalian cells infected by *P. aeruginosa* [10, 11]. In addition, it produces other pro-inflammatory cytokines IL-1β, IL-6, and IL-18 through the nuclear factor κB (NF-κB) pathway [12, 13]. Among the sources of infectious toxicity of *P. aeruginosa*, we selected ExoS as the target strain here among T3SS because ExoS is a virulence factor with these main characteristics.

The polyphenol quercetin (3,3¢,4¢,5,7-pentahydroxyflavone) is a flavonoid found in many fruits (cranberries, chokeberries, capers, apples), leaves, grains, and vegetables (asparagus) and medicinal herbs such as lovage, cilantro, radish leaves, and dill. Typically, kale and red onions are common foods containing appreciable amounts of quercetin [14, 15]. Quercetin has a bitter flavor and is used as an ingredient in foods, beverages, and dietary supplements [16]. This compound reportedly has pharmacological effects, such as anti-oxidant [17], anti-inflammatory [18, 19], anti-virus [20], and anti-diabetic effects [21], can be found in therapeutic cardiovascular agent [22], and even has anti-tumor activities [17, 23, 24]. However, with regard to the ExoS of *P. aeruginosa*, an inhibitory effect of quercetin has not been reported.

Therefore, in this experiment, the effect of quercetin on the inhibitory activity of *P. aeruginosa* ExoS secretion and *P. aeruginosa* T3SS expression levels were investigated.

## Materials and Methods

### Quercetin

Quercetin was supplied as a purified compound (code No. NPFL10051.1) from the *Styphnolobium japonicum* (L.) Schott in the Korea Plant Extract Bank (https://extract.kribb.re.kr) at the Korea Research Institute of Bioscience & Biotechnology (KRIBB, Korea). The mass spectrum of compound was displayed as characteristic fragments of quercetin at *m/z* 151 (C_7_H_3_O_4_) and 179 (C_8_H_3_O_5_) generated from the predominant ion *m/z* 301 (C_15_H_9_O_7_, [M–H]^–^) in negative mode (Fig. S1.1) [25]. Also, quercetin was elucidated by comparison with its spectroscopic data (^1^H, ^13^C NMR, and HR-ESI-QTOF/MS) and reported a literature [26]. The structural information of authentic compound was listed as a supplementary data (Fig. S1.2 and S1.3).

### Bacterial Strains

Chromosomal mutants were all derived from the same parental PAK (*P. aeruginosa* strain K; wild-type strain) strain [27-29] and were generated by the allelic exchange method. All strains and plasmids used in this study are listed in Table 1. All strains were either in PAK genetic backgrounds or were previously described [27, 30]–except for the PAK ExoST::Ω, PAK ExoST::Ω/ExoS-FLAG strains, which were constructed for these studies. PAK ExoST::Ω was PAK ExoST::Ω/pHW0225 strains were PAK ExoST harboring an ExoS–Flag fusion construct were grown in L broth in the presence (+) of 1 mM EGTA for 12 h and cultured. Under all conditions, effector secretion was triggered by removing calcium from the medium through the addition of EGTA (1 mM final concentration).

### Bacterial Culture and Growth Curve

All bacterial strains were grown overnight in Luria-Bertani (LB) media with 1 mM EGTA at 37°C on a rotary shaker if not otherwise specified. Bacterial cells were harvested at 12,000 g at 4°C for 10 min after being left overnight in LB broth culture. Antibiotics were used at the following concentrations; spectinomycin (Sp; 200 μg/ml, Cat. No. S9007-5G), streptomycin (Sm; 200 μg/ml, Cat. No. S6501-5G), gentamycin (Gm; 150 μg/ml, Cat. No. G1264, Sigma-Aldrich, USA) and carbenicillin disodium (Cb; 150 μg/ml, Cat. No. 2485-1G, BioVision, USA) [29-31]. Overnight cultures of an untreated *P. aeruginosa* PAK strain and *P. aeruginosa* treated with a 20 μM concentration of quercetin were re-inoculated and diluted at 1:1,000 in fresh LB broth. Growth of the bacteria was determined at OD 600 using an ultraviolet–visible (UV/VIS) spectrophotometer (Cat. No. Optizen 2120UV, Mecasys Co., Ltd., Korea) [32].

### ExoS-FLAG Enzyme-Linked Immunosorbent Assay (ELISA)

**Fig. 1:**
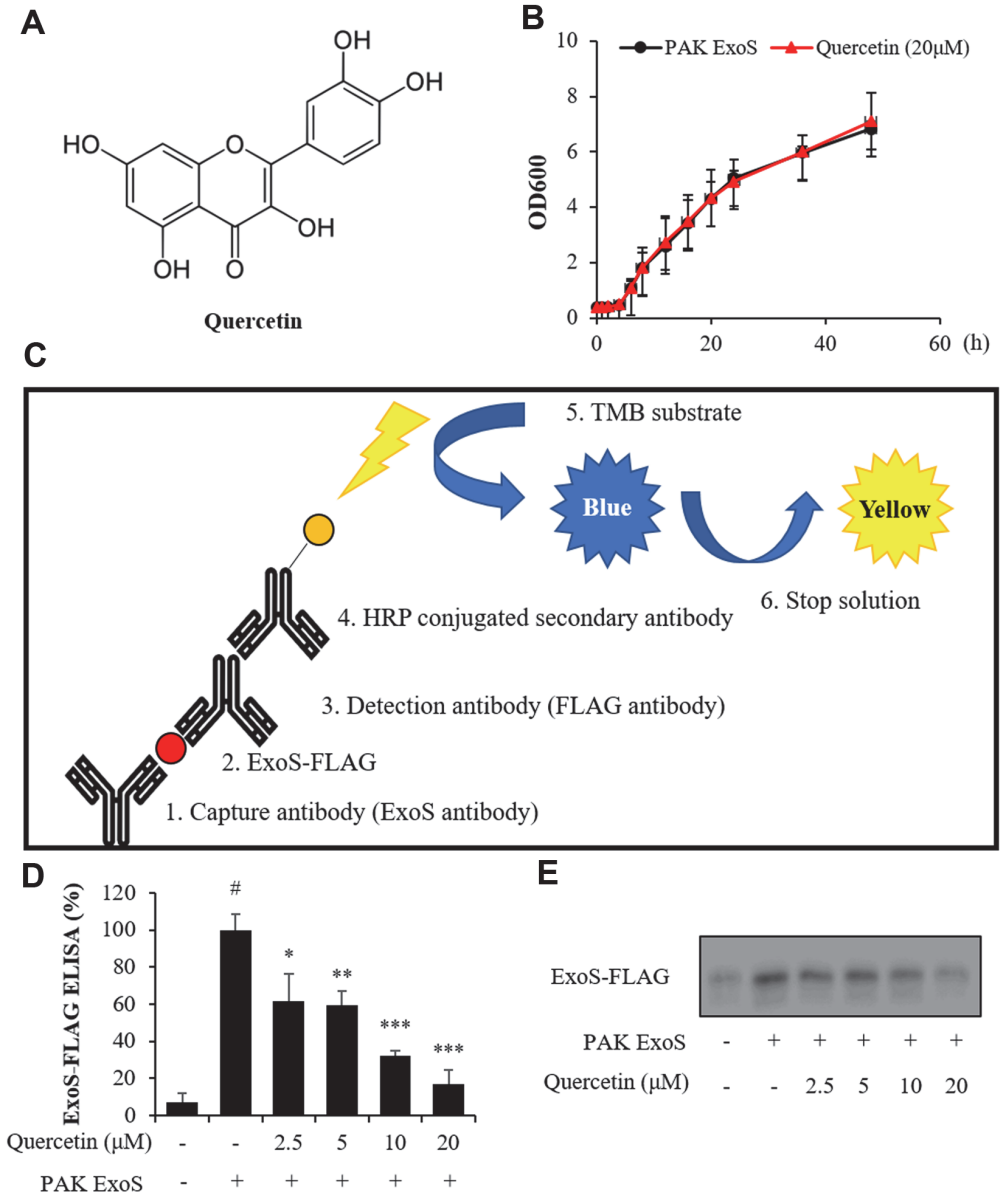
Effect of quercetin on the bacterial growth and on ExoS-FLAG produced and secreted. (**A**) The compound structure of quercetin. (**B**) The effect of quercetin on the growth of *P. aeruginosa* was determined by the bacterial growth curve. PAK, *P. aeruginosa* wild-type strain. (**C**) The principle of ExoS-FLAG ELISA is illustrated. (**D**) Overnight culture supernatant of *P. aeruginosa* strains treated with quercetin (2.5, 5, 10, and 20 μM) as used in the ExoS-FLAG ELISA. (**E**) Lysed proteins of *P. aeruginosa* strains are loaded via 10% SDS-PAGE. Negative control (-), PAK ExoST::Ω; PAK ExoS; Positive control (+), PAK ExoST::Ω/pHW0225, After PAK ExoST was double mutated, the FLAG attached to ExoS was reinserted; #;significantly different from the normal control group, *p* < 0.01. *, **, *** significantly different from the positive control at *p* < 0.05, *p* < 0.01, and *p* < 0.001, respectively.

To detect ExoS secretion, the secreted ExoS-FLAG amounts in bacterial overnight culture supernatants supplemented with quercetin and 1 mM of EGTA (type III inducing condition) were determined using an ExoS-FLAG sandwich ELISA system (Fig. 1C) designed according to a previous study [33]. An anti-ExoS antibody as a capture antibody was commissioned by Koma Biotech (Korea), Inc. and produced by Prosci, Inc. (USA). The amino acid sequence of ExoS was useful information with regard to C- KPERSGEVQEQDVRLRMRGLDLA. The ExoS amino acid sequence was diluted to 1:5,000 in a carbonate-bicarbonate buffer (0.05 M, pH 9.6), coated into 96-well microplates at 4°C, and kept overnight. The plates were incubated with a blocking buffer (2% bovine serum albumin (BSA) in phosphate-buffered saline (PBS)) for 1 h, after which they were washed and a bacterial supernatant was added to each well. After incubation for 2 h, the plates were washed and mouse anti-FLAG antibody (Anti-DDDDK antibody; Cat. No. ARG62342, Arigo Biolaboratories, Taiwan), a detection antibody diluted to 1:5,000 in 1% BSA (Bovine Serum Albumin; Cat. No. BSAS0.1, Bovogen Biologicals Pty. Ltd., Australia) in PBS (assay diluent) was added to each well. After incubation for 1 h, the plates were washed, and horseradish peroxidase (HRP)-conjugated goat anti-mouse immunoglobulin G (IgG) diluted to 1:2,000 in an assay diluent was then added to each well for 1 h. After incubation, the plates were washed and a substrate solution was added to each well. Lastly, the color reaction of the plates was stopped with H_2_SO_4_ and the absorbance was determined at 450-570 nm using a Spark 10 M multimode microplate reader instrument. All steps except for the incubation of the capture antibody were performed at room temperature.

### Cell Culture

The H292 cell line (ATCC; American Type Culture Collection, USA) consists of human epithelial cells derived from human lung carcinoma. H292 cells were cultured in the RPMI 1640 medium (Cat. No. LM 011-01, Welgene, Korea) supplemented with 10% fetal bovine serum (FBS; Invitrogen, USA) and antibiotics (1% penicillin-streptomycin) and incubated at 37°C in a 5% CO_2_ incubator.

### MTT Assay

Cell viability was measured via a 3-(4,5-dimethylthiazol-2-yl)-2,5-diphenyl tetrazolium bromide (MTT) assay. H292 cells were seeded into 96-well plates (SPL, Korea) at a concentration of 1 × 10^4^ cells/well. After incubation for 5 h, the cells incubated with various concentrations of quercetin for 20 h. In the infection cases, H292 cells were infected by moment of infection (MOI) 100 of the PAK ExoS strain after a quercetin treatment for 1 h. Subsequently, 5 μl of a 5 mg/ml MTT solution (Amresco, LLC, USA) was added to each well for 4 h. The supernatant was removed, and 100 μl of dimethyl sulfoxide (DMSO) was added to each well to dissolve the formazan crystals.

### Cytokine ELISA Assay

H292 cells were seeded into 12-well plates at a concentration of 4 × 10^6^ cells/well and cultured overnight at 37°C. After incubation, the media in the plates were changed to RPMI 1640 without supplementation, and the cells were pretreated with various concentrations of quercetin for 1 h. The H292 cells were then infected by MOI 100 of the PAK ExoS strain at 37°C for 5 h. Cytokines of the culture supernatant of the infected cells were measured using ELISA kits (IL-18 set, Cat. No. DY318-05, R&D system, Minneapolis, USA; IL-1β set, Cat. No. 557953; IL-6 set, Cat. No. 555220; BD Biosciences, USA). Each experiment was conducted according to the manufacturer’s protocol.

### Real-Time Polymerase Chain Reaction (PCR) Analysis

Briefly, H292 cells were seeded into 12-well plates at a concentration of 4 × 10^6^ cells/well and cultured at 37°C overnight. The cells were treated with quercetin for 1 h and then PAK ExoS for 5 h. In contrast, bacterial cells were grown overnight at 37°C in a rotary shaker and re-inoculated in fresh LB broth supplemented with quercetin and 1 mM of EGTA for 4 h. After all of the steps, the H292 cells and bacterial cells were harvested. The total RNA was isolated using the TRIzol Reagent (Invitrogen; Thermo Fisher Scientific, Inc., USA). Synthesis of the complementary DNA was performed using a ReverTra Ace qPCR RT Master Mix kit (Cat. No. FSQ-301, Toyobo, Japan). Relative mRNA levels were calculated using the comparative threshold cycle 2-ΔΔCq method and normalized to that of GAPDH and S16. The cDNA was used for real-time PCR with primers (Table 2, Bioneer, Korea). This experiment was conducted using the SYBR Green PCR Master Mix (KAPA Bio-systems, USA).

### Western Blot Analysis

For ExoS-FLAG detection, the PAK ExoS strains (Table 1) was grown overnight in LB broth supplemented with quercetin and 1 mM of EGTA at 37°C in a rotary shaker. Bacterial cells were then harvested into 1.5-ml tubes and lysed using a diluted 5× bacterial protein extraction reagent (Cat. No. AKR-180, Cell Biolabs, Inc., USA) for protein extraction. The bacterial protein quantity was measured using a Pierce BCA Protein assay kit (Cat. No. 23225, Thermo Fisher Scientific Inc.).

Equal amounts of protein were loaded and separated using 10% sodium dodecyl sulfate polyacrylamide gel electrophoresis (SDS-PAGE). Loaded proteins were transferred to a hydrophilic polyvinylidene fluoride membrane (PVDF; EMD Millipore, USA). Each membrane was blocked with 5% skim milk in tris-buffered saline with Tween (TBS-T) for 1 h at room temperature. The primary antibodies used ExoS-FLAG (Anti-DDDDK antibody; Cat. No. ARG62342, Arigo Biolaboratories, Taiwan, ROC), β-actin (MA5-15739), anti-phospho IKKα/β (2697S), anti-IKKβ (8943S), anti-IκBα (MA5-15132), anti-phospho-IκBα (MA5-15087, Thermo Fisher Scientific Inc., USA), anti-p65 (8242S), anti-phospho-p65 (3033S), anti-caspase-1 (3866S), anti-NLRP3 (13158), and anti-NLRC4 (124215S, Cell Signaling Technology, USA), were was diluted to 1:1,000 in 5% skim milk in TBS-T, added to the membrane rack, and then incubated overnight at 4°C. After incubation, the membranes were washed with TBS-T and incubated with secondary antibody HRP-conjugated anti-mouse IgG (Cat. No. sc-2005, Santa Cruz Biotechnology Inc., USA) and anti-rabbit IgG (Cat. No. 111-035-003, Jackson Immune Research Laboratories, Inc, USA) diluted to 1:5,000 in 5% skim milk in TBS-T for 1 h at room temperature. The protein bands were visualized using a chemiluminescence kit (ECL; Cat. No. 32106, Thermo Fisher Scientific Inc.) and a luminescent image analyzer (LAS-4000, Fujifilm, Japan).

### Protein Analysis for p65 Translocation via Nucleus Fraction Western Blotting

We confirmed the p65 level when NF-κB was activated by measuring p65 in the nucleus. This process was performed using a nucleus extract kit (Nucleus extract kit, Cat. No. 2900, EMD Millipore, USA). After creating a cell pellet, foregoing the western blot step, we obtained cytosol protein using a cytosol extract solution and washed this once with cold PBS. Another pellet was created with a centrifuge and with the nucleus protein fraction via a nucleus extract solution. The electrophoresis step was identical to the western blotting method for the whole protein, and we detected the p65 antibody using an antibody for rabbit (Horseradish peroxidase (HRP)-conjugated goat anti-rabbit immunoglobulin G (IgG), Cat. No. 111-035-003, Jackson, USA).

### Statistical Analysis

The data are expressed as the mean ± SEM. Statistical significance was determined using a two-tailed Student’s *t*-test. A value of *p* < 0.05 was considered to indicate a statistically significant difference.

## Results

### Effects of Quercetin on Bacterial Growth

It is important to ensure when the material is processed that it will affect bacterial growth. Thus, experiments are needed to determine whether a quercetin treatment has an effect on bacterial growth. To confirm the effect of quercetin on bacterial growth here, the growth of the *P. aeruginosa* PAK strain with quercetin was determined at an optical density [34] of 600 for 48 h by a spectrophotometer. As a result, there was no effect on the growth rate compared to untreated PAK (Fig. 1B). Therefore, this finding indicates that the data presented later is not related to the growth of *P. aeruginosa*.

### Quercetin Reduced the Secretion of ExoS-FLAG from the *P. aeruginosa*

The ExoS of T3SS (ExoS, ExoT, ExoU, ExoY) has been reported as one of the most important virulence factors of *P. aeruginosa* [35]. In order to determine whether quercetin inhibits the secretion of ExoS, which is a virulence factor, an ExoS-Flag ELISA system was built in-house. To measure the amount of secreted ExoS, an ExoS-FLAG ELISA was conducted. The principle of the ExoS ELISA is illustrated in Fig. 1C. Quercetin has a definite inhibitory effect of more than 40% in 2.5 μM and 5 μM and 50% in 10 and 20 μM (Fig. 1D). We also conducted a western blotting analysis to detect any remaining ExoS in the bacteria. As a result, the expression of ExoS in bacteria was found to be inhibited (Figs. 1D and S2). Quercetin has the effect of inhibiting ExoS as identified in the ExoS-FLAG ELISA test. Thus, these results confirmed that quercetin inhibits both the secretion and expression of ExoS. Through this result, we considered that ExoS was related to the transcription of ExsCEBA controlling the expression of ExoS, or the expression of *popB/D* controlling the secretion of ExoS.

### Quercetin Reduced the Expression of ExoS, *popB*, *popD*, *pscF*, and *pcrV* in *P. aeruginosa*.

**Fig. 2:**
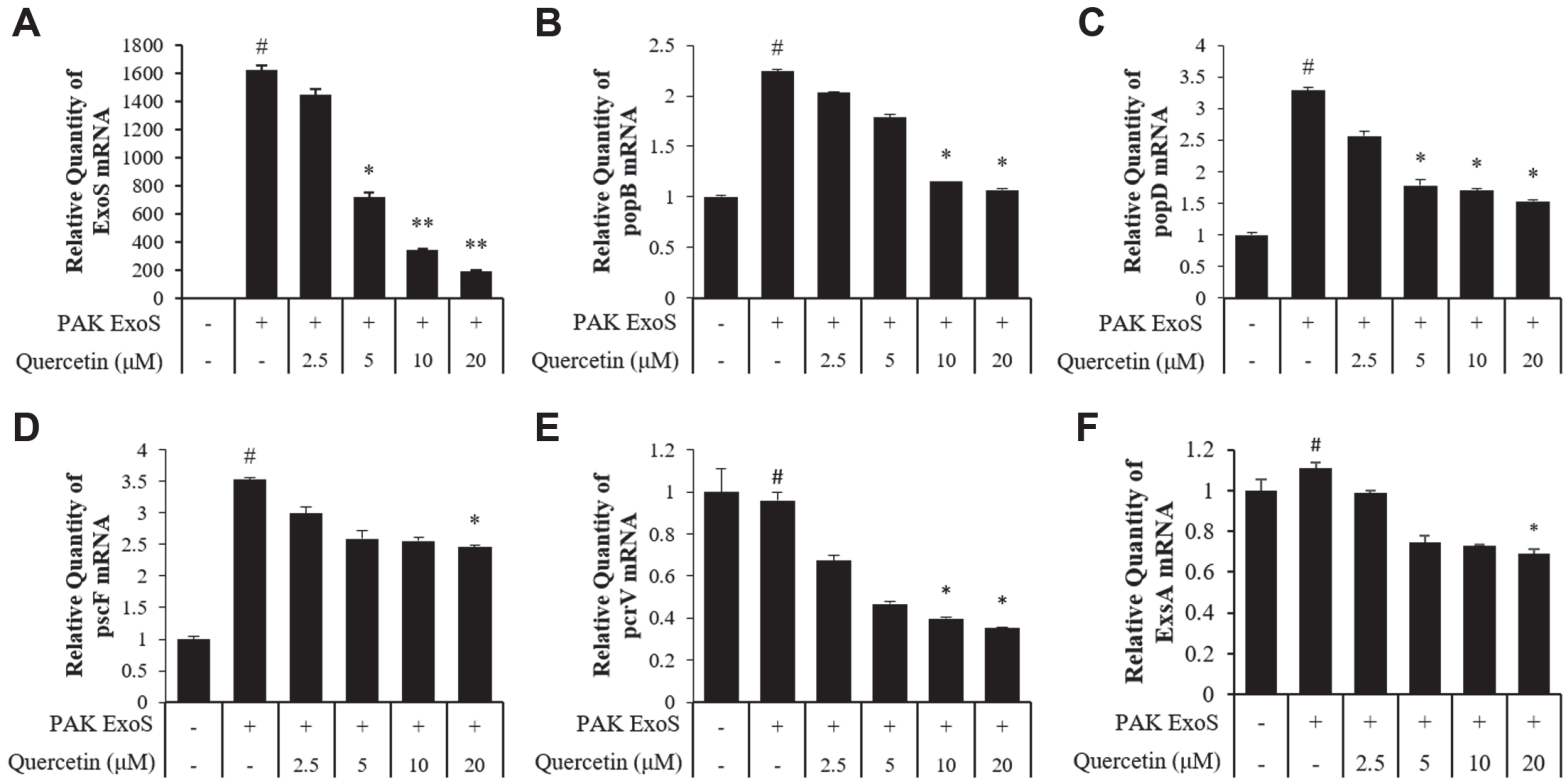
Effect of quercetin on type III secretion system regulators and effector protein in *P. aeruginosa*. ΔST and PAK ExoS strains were grown overnight at 37°C and re-inoculated in fresh LB broth supplemented with quercetin (2.5, 5, 10, and 20 μM) for 4 h. The expression levels of (**A**) ExoS of exoenzyme, (**B**) *popB*, (**C**) *popD*, (**D**) *pscF*, and (**E**) *pcrV*, and the needle protein of T3SS ExoS were quantified by quantitative real-time PCR. (**F**) The expression of ExsA of the transcription factor of T3SS was quantified in the same manner. Expression analysis of the regulators and effector protein indicated normalization with the 16S rRNA gene. ΔST (-), PAK ΔST; PAK ExoS (+), PAK ΔST/pUCP18 PAK ExoS. #Significantly different from the normal control group at *p* < 0.05; *significantly different from the PAK ExoS group at *p* < 0.05.

An important virulence factor of PAK is the T3SS, a hollow molecular needle that transfers effector toxins directly from the bacterium into the host cell cytosol [7]. Proteins involved in these needles include PopB, PopD, PscF and PcrV, and there is a transcription factor, ExsA. To evaluate the expression levels of the effector protein and regulators of T3SS at the mRNA level, quantitative real-time PCR of factors related to T3SS was conducted. This process showed that, the expression of ExoS was inhibited and that the expression levels of the needle proteins PopB, PopD, PscF, and PcrV were also suppressed (Figs. 2A-2E). Subsequently, the transcription factor ExsA was found to have a dose dependent inhibition effect (Fig. 2F). These results show that quercetin ultimately reduces the secretion of ExoS by influencing both the expression of ExoS and the secretion system. Through these results, it can be expected that quercetin can effectively suppress inflammatory reactions by host cells by reducing the amount of ExoS secreted by P.aeruginosa.

### Cytotoxic Effects of Quercetin in H292 Cells

**Fig. 3:**
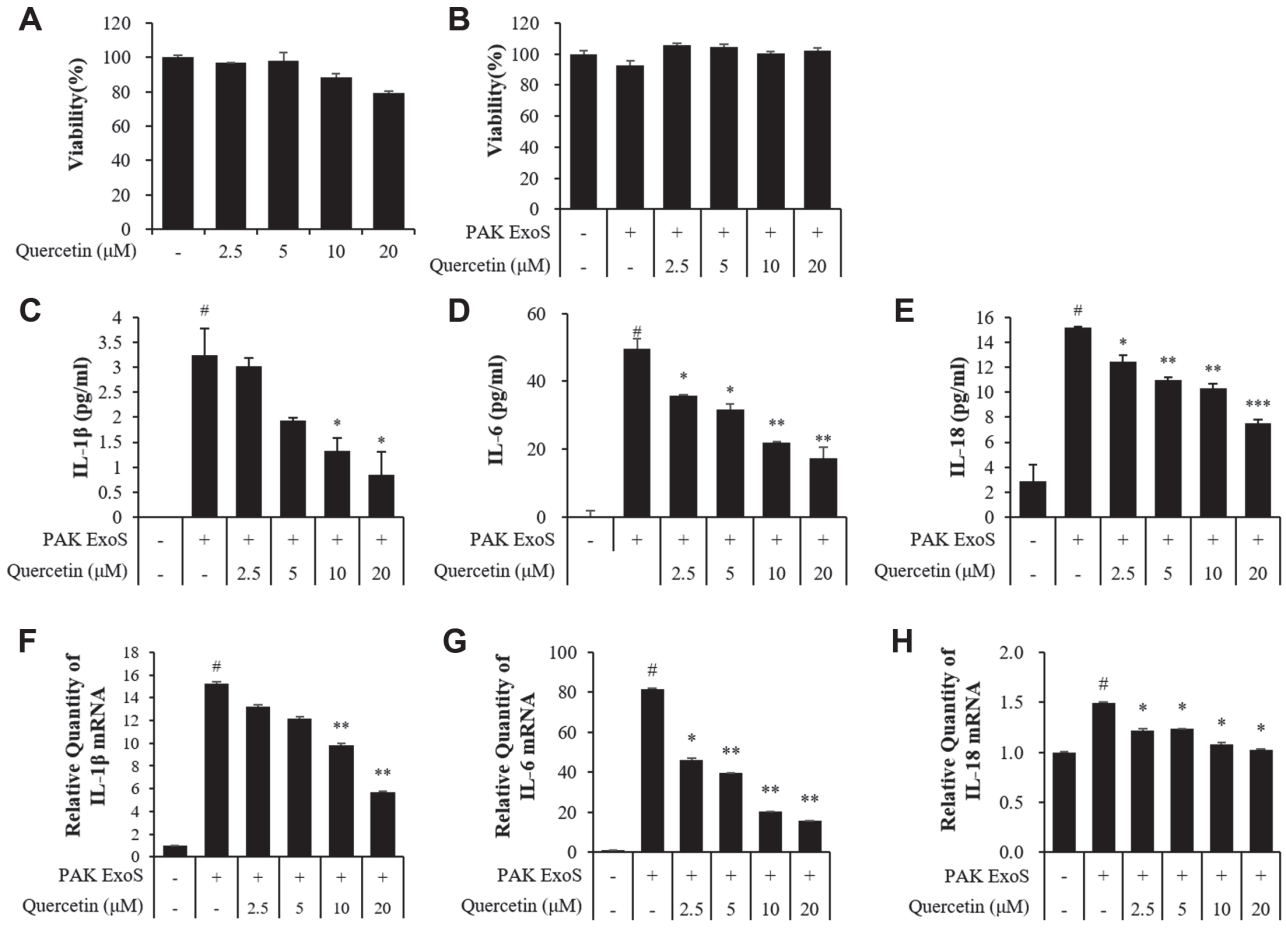
Cytotoxicity of quercetin in uninfected or infected H292 cells. (**A**) H292 cells were incubated with quercetin (2.5, 5, 10, and 20 μM). (**B**) With regard to infection, H292 cells were infected by MOI 100 of the PAK ExoS strain after a quercetin treatment (2.5, 5, 10, and 20 μM) for 1 h. The cytotoxicity of quercetin was evaluated by a MTT assay. (**C**) IL-1β, (**D**) IL-6, and (**E**) IL-18 protein levels of pro-inflammatory cytokines were evaluated by ELISA and (**F**) IL-1β, (**G**) IL-6, and (**H**) IL- 18 mRNA levels were evaluated by real-time PCR. PAK ExoS; PAK ΔST/pUCP18 PAK ExoS. #Significantly different from the normal control group, *p* < 0.01. *, **, significantly different from the PAK ExoS group, *p* < 0.05, and *p* < 0.01gy, respectively.

MTT assays were used to identify the toxicity of quercetin on H292 cells. When quercetin (2.5, 5, 10, and 20 μM) alone was used, all concentrations of quercetin showed no toxicity on H292 cells (Fig. 3A). likewise when quercetin was exposed to *P. aeruginosa* PAK ExoS infected H292 cells, it was no toxicity of the *P. aeruginosa* PAK ExoS strain (Fig. 3B). Therefore, these results suggest that quercetin is non-toxic and in fact has an anti-toxic effect.

### Quercetin Reduced Pro-Inflammatory Cytokines in *P. aeruginosa*- Infected H292 Cells

Among the cytokines, IL-1β, IL-6, and IL-18 are known to be very important cytokines that respond to a PAK infection [36-38]. We aimed to identify how the expression and secretion levels of ExoS reduced by quercetin affected infected H292 cells. Therefore, the secretion and expression of cytokines induced by ExoS were verified with ELISA and real-time PCR, respectively (Figs. 3C-3H) [39]. Quercetin did not affect the cytokine products of H292 cells of IL-1β, IL-6, and IL-18 when used alone as a treatment. This was confirmed by real-time PCR (Figs. 3F-3H). As a result, the treatment of quercetin was found to decrease the secretion and expression levels of cytokines (IL-1β, IL-6, and IL-18), which also suggests that the decreased levels of cytokines were due to the decreased ExoS secretion levels.

### Effect of Quercetin on the NF-κB Pathway in *P. aeruginosa*-Infected H292 Cells

**Fig. 4:**
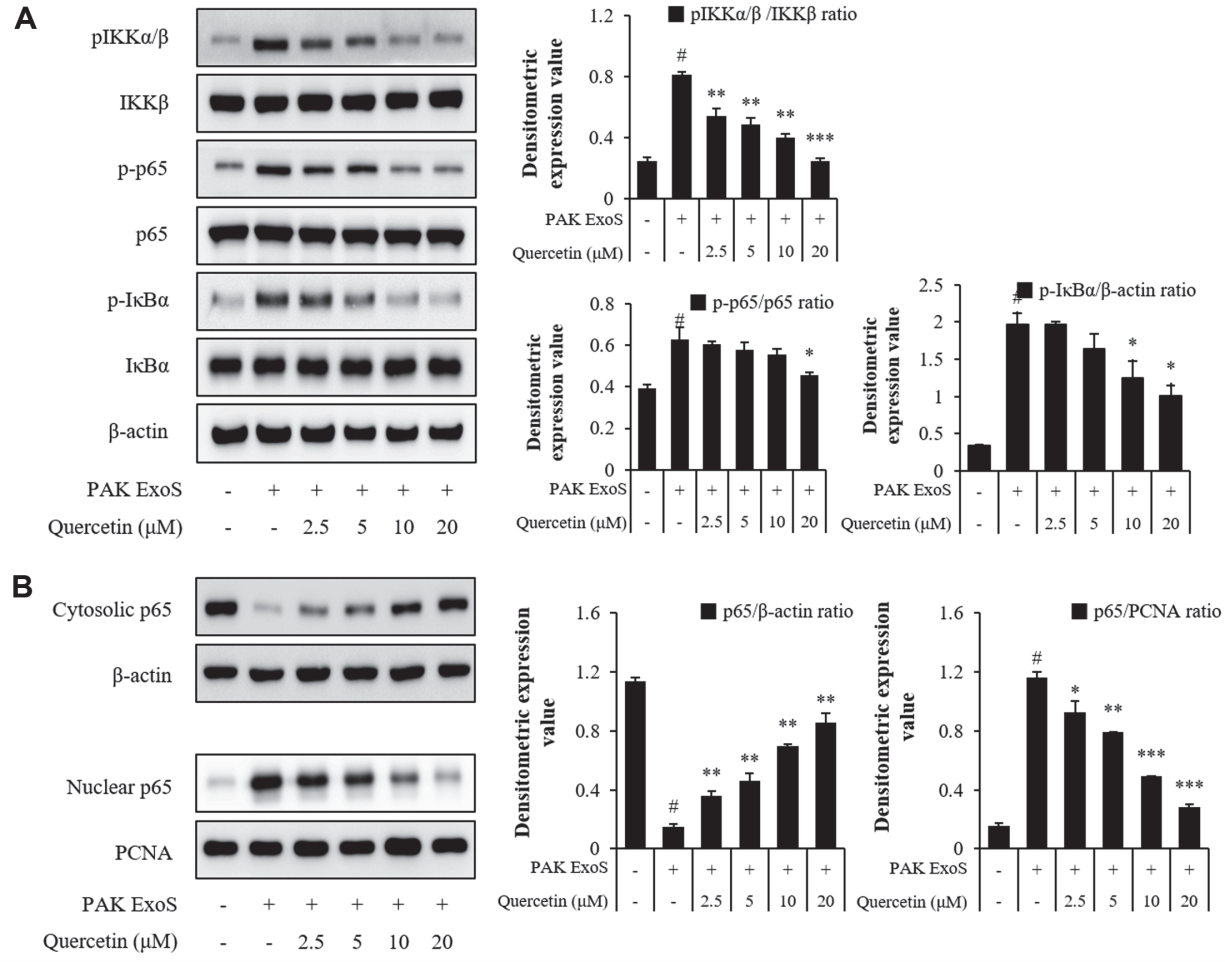
Effects of quercetin on *P. aeruginosa*-induced NF-κB activation in H292 cells. H292 cells were treated with quercetin and PAK ExoS for 1 h. The expression of the NF-κB pathway was evaluated by western blotting. PAK ExoS; PAK ΔST/pUCP18 PAK ExoS.

NF-κB activation in cells promotes the production of various cytokines, such as IL-1β, IL-6, and IL-18, which serve as growth factors for the transformation [40, 41]. Previously, the effects of caspase-1 pathways were checked and, the results of the NF-κB pathways in cells infected with *P. aeruginosa* were tested. IκB and the phosphorylation of p65 were checked through the western blot analyses, which showed that phosphorylation in both targets decreased compared to the normal form. Therefore, it was confirmed that the additional NF-κB pathways due to infection of *P. aeruginosa* were inhibited through quercetin. (Figs. 4 and S3.1-S3.7) In addition, to confirm the decreased phosphorylation of p65, the level of p65 translocated into the nucleus was measured; these findings confirmed that the effect was evident when alizarin was treated for 30 minutes (Figs. 4B and S3.8-S3.11). From the results overall, it is likely that quercetin reduced cytokine production and secretion levels by inhibiting the phosphorylation of the p65 factor by ExoS.

### Quercetin Inhibits the Induction of the NLRP3/NLRC4/caspase-1 Pathway by *P. aeruginosa* Infection

**Fig. 5:**
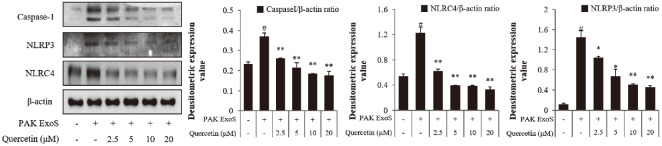
Effect of quercetin on the *P. aeruginosa*-induced NLRP3/NLRC4/Caspase-1 pathway in H292 cells. H292 cells were treated with quercetin and PAK ExoS for 2 h. The expression levels of NLRP3, NLRC4, and Capase-1 were evaluated by western blotting. PAK ExoS; PAK ΔST/pUCP18 PAK ExoS.

Pro-inflammatory cytokines induced by ExoS were confirmed to be reduced by quercetin. Therefore, western blots were utilized to determine the expressions of NLRP3, NLRC4, and caspase-1, which induce pro-inflammatory cytokines. As a result, the increased caspase-1 level in infected cells was reduced when quercetin was utilized, and NLRC4 showed the same result (Figs. 5 and S4.1-S4.4). These results suggest that quercetin reduces ExoS and further affects the NLRP3, NLRC4 and caspase-1 pathways.

## Discussion

Multi-drug resistant strains of *P. aeruginosa* continue to present a persistent and critical danger to immunocompromised patients, particularly in intensive care settings [42]. These problems can be resolved with the development of therapeutic agents such as blockers of *P. aeruginosa* infection and virulence. *P. aeruginosa* has a variety of systems necessary for infection, and T3SS can act as a major virulence factor during various *P. aeruginosa* infections. T3SS activation requires specific environments, such as contact with host cells and a low concentration of calcium [33]. Previously, we demonstrated that ExoS can cause an inflammatory response and improve clearance in the lungs in vitro and vivo [27]. It is also well known that quercetin is a useful substance for various diseases [43-45]. However, it is not known whether quercetin prevents disease by inhibiting the expression of ExoS due to infection of the host via T3SS of *P. aeruginosa*. Therefore, we aimed to elucidate the mechanism of the anti-inflammatory activity of quercetin associated with the ExoS of *P. aeruginosa*.

Among the toxic substances secreted by T3SS, ExoS has a structure similar to that of ExoT and has functions similar to those of ADPRT and GTPase [1]. However, ExoS has higher toxicity than the ExoT and is expressed in some *P. aeruginosa* clinical isolates [46, 47]. Conversely, because the ExoT is expressed in most *P. aeruginosa* clinical isolates, this experiment used a strain into which a plasmid was inserted so that only ExoS can be expressed while knocking out ExoT.

T3SS is transcribed through the ExsCEBA site, which can be considered as a T3SS regulator. The secretion activity is carried out by ExsC, ExsD, ExsE, and ExsA [4]. We investigated the inhibitory effects of quercetin on the expression of ExsA, the transcriptional activator of T3SS operons, among others, at the mRNA level. It was found that quercetin decreased the expression of ExsA, with ExoS production of T3SS followed by ExsA also decreased. These results indicate that quercetin has the effect of suppressing the overall expression of T3SS.

The needle proteins, which act as a translocator so that the secretory toxins of T3SS can be secreted, consists of *pscF*, *pcrV*, and *popB/D*. *pscF* serves as a major component for needle proteins among them, while *popD* is responsible for direct insertion into host cells. Additionally, *popB* is responsible for assisting in the insertion of *popD* [48, 49]. Therefore, we investigated whether quercetin affects the expression levels of *pscF* and *popD*, which account for the most important needle protein region. The results show that quercetin reduces the expression of the T3SS needle protein, which can be directly toxic to host cells by injecting the generated secretory toxins. Through this result, we demonstrate that quercetin has an inhibitory effect on the virulence factors of *P. aeruginosa*. Host cells infected with *P. aeruginosa* produce cytokines such as IL-1β and IL-18 resulting from the activation of caspase-1, and additionally activates inflammatory signals, such as NF-κB pathways, by injecting ExoS [27]. Caspase-1 has proteolytic activity for the precursors of IL-1β and IL-18 and converts them into IL-1β and IL-18. Also, after caspase-1 is activated, it causes cell death by proptosis [50, 51]. Therefore, the secretion and expression levels of cytokines (IL-1β, IL-6, and IL-18) induced by ExoS were verified with ELISA and real-time PCR assessments, respectively. As a result, the treatment with quercetin was found to decrease the secretion and expression levels of the cytokines, which suggests that the reduced cytokines in H292 cells are due to the decreased ExoS secretion by quercetin. According to earlier work, caspase-1 is activated by NLRC4 or NLRP3 forming an inflammasome [52]. Quercetin inhibits the NLRC4 inflammasome and caspase-1 induced by *P. aeruginosa* according to a western blot analysis. Additionally, further research is needed on whether ExoS as reduced by quercetin also affects NLRP3. All of these findings suggest that quercetin alleviates cytotoxicity caused by infection by reducing the generation of ExoS, and it is expected that its effect on the ExoS of T3SS will prevent *P. aeruginosa* from developing lung-related diseases caused by the infection of host cells. It is thought that in vivo experiments will be needed to support this finding.

**Fig. 6:**
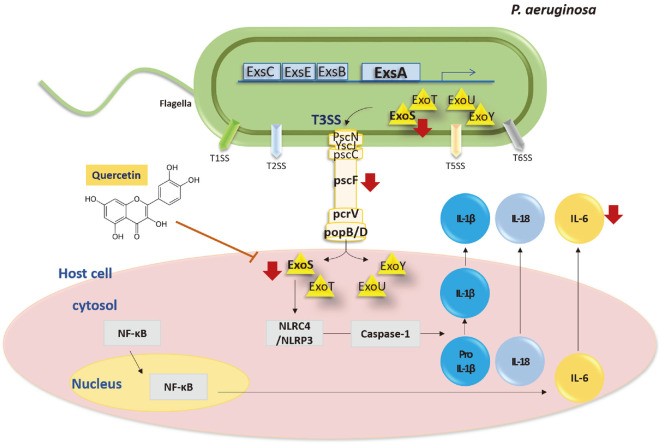
Mechanism of quercetin on ExoS in *P. aeruginosa* and infected H292 cells. ExoS production of T3SS followed by ExsA decreased and the expression levels of the T3SS (*popB*, *popD*, *pscF*, and *pcrV*) needles also decreased, after which the injection of ExoS from P. aerugnosa to H292 cells was suppressed by quercetin. The expression of the NLRP3/NLRC4/Caspase-1 pathway and the production of pro-inflammatory cytokines (IL-1β, IL-18, IL-6) induced by ExoS are also decreased. PAK ExoS; PAK ΔST/pUCP18 PAK ExoS.

In summary, quercetin has been shown to decrease ExoS production. It also inhibits the expression of needle proteins and reduces the secretion of ExoS. In addition, it was confirmed that there is the effect of a reduction of pro-inflammatory cytokines (IL-6, IL-1β, and IL-18) upon a reduction of the activation of the NLRP3/NLRC4/caspase-1 pathway in H292 cells (Fig. 6). However, the suppression of ExoS is not due to ExsA. Thus, further study of another factor in which quercetin affects the suppression of ExoS is likely to be needed.

In conclusion, quercetin not only reduces the generation of ExoS but also inhibits the expression of the needle protein, reducing the secretion of ExoS. Through this, it was confirmed that quercetin decreases the activity of NF-κB by inhibiting the production of cytokines and decreases the activation of the caspase-1 pathway. In the future, we expect to be able to demonstrate the efficacy of quercetin by elucidating deeper mechanisms/pathways. Furthermore, the activation of caspase-1 pathways was decreased within eukaryotic cells. Therefore, it is expected that quercetin can be used as a treatment given its anti-toxic, anti-inflammatory activity even in lung diseases resulting from infection with *P. aeruginosa*.

## Supplemental Materials

Supplementary data for this paper are available on-line only at http://jmb.or.kr.

## Figures and Tables

**Table 1:** List of all strains and plasmids used in this study.

Strains	Description	Antibiotics	Source or reference
PAK (WT)	Wild-type strain		From Dr. Ha (Korea university)
PAK exoST::Ω (Negative control; -)	PAK with chromosomal disruption of the exoS and exoT loci with Ω cassette	Sp^200^, Sm^200^, Gm^200^, Cb^150^	From Dr. Ha
PAK exoST::Ω/pHW0225 (Positive control; +)	After PAK ExoST double mutated, the FLAG attached to ExoS is reinserted.	Sp^200^, Gm^200^, Cb^150^	From Dr. Ha
PAK ΔST (ΔST)	Double effector mutant; functional needle and translocon apparatus without known effectors		From Dr. Ha
PAK ΔST/pUCP18 PAK ExoS (PAK ExoS)	PAKΔST with exoS of pUCP18	Cb^150^	From Dr. Park (Korea Research Institute of Bioscience and Biotechnology)

**Table 2:** Sequence of primers used for real-time PCR.

*P. aeruginosa* 16S	
Sense	5’-CAA AAC TAC TGA GCT AGA GTA CG-3’
Anti-sensef	5’-GCC ACT GGT GTT CCT TCC TA-3’
*P. aeruginosa* ExoS (113bp)	
Sense	5’-CAG GCT GAA CAG GTA GTG AA-3’
Anti-sense	5’-TTC AGG GAG GTG GAG AGA TA-3’
*P. aeruginosa* *popB* (	
Sense	5’-GCG CTT CGA CGC TGT TGT-3’
Anti-sense	5’-TTC TTC CGA CTC CCT GAT CTT CT-3’
*P. aeruginosa* *popD*	
Sense	5’-GAA GAC CCT GCA GAA GAA CA-3’
Anti-sense	5’-ACC TTG CCG ACG ATC TTG-3’
*P. aeruginosa* *pscF*	
Sense	5’-AAC GCA GCG AAC AAG GA-3’
Anti-sense	5’-GTT GTA GAT GAC CGA CCA CTT-3’
*P. aeruginosa* *pcrV*	
Sense	5’-GAT CGA CGC TGG CGG TAT-3’
Anti-sense	5’-TCA TCG CTG AGG CCC TTG-3’
*P. aeruginosa* ExsA	
Sense	5’-AAG GAG CCA AAT CTC TTG-3’
Anti-sense	5’-CTT GTT TAC CCT GTA TTC G-3’
Human β-actin	
Sense	5’-CAT GTA CGT TGC TAT CCA GGC-3’
Anti-sense	5’-CTC CTT AAT GTC ACG CAC GAT-3’
Human IL-1β	
Sense	5’-GGA CAA GCT GAG GAA GAT GC-3’
Anti-sense	5’-TCT TTC AAC ACG CAG GAC AG-3’
Human IL-18	
Sense	5’-GCT TGA ATC TAA ATT ATC AGT C-3’
Anti-sense	5’-GAA GAT TCA AAT TGC ATC TTA T-3’
Human IL-6	
Sense	5’-GAC AGC CAC TCA CCT CTT CA-3
Anti-sense	5’-AGT GCC TCT TTG CTG CTT TC-3’
