# Supplementary material for: Quercetin Attenuates the Production of Pro-Inflammatory Cytokines in H292 Human Lung Epithelial Cells Infected with Pseudomonas aeruginosa by Modulating ExoS Production
Source: J Microbiol Biotechnol. 2023 Jan 27;33(4):430–40. doi: 10.4014/jmb.2208.08034 (PMC10164726; doi:10.4014/jmb.2208.08034)
Supplement: Supplementary file 1 [file jmb-33-4-430-supple.pdf]

J. Microbiol. Biotechnol.
https://doi.org/10.4014/jmb.2208.08034

Supplementary Figures

Quercetin Attenuates the Production of Pro-Inflammatory Cytokines in H292 Human Lung Epithelial Cells Infected with *Pseudomonas aeruginosa*, by Modulating ExoS Production

Hye In Ahn[3#], Hyun-Jae Jang[1], Ok-Kyoung Kwon[1], Jung-Hee Kim[1], Jae-Hoon Oh[1,2], Seung-Ho Kim[1,2], Sei-Ryang Oh[1], Sang-Bae Han[2], Kyung-Seop Ahn[1*], and Ji-Won Park[1,4*]

[1]*Natural Medicine Research Center, Korea Research Institute of Bioscience and Biotechnology, 30 Yeongudanji-ro, Ochang-eup, Cheongwon-gu, Cheongju-si, Chungbuk 28116, Republic of Korea*

[2]*College of Pharmacy, Chungbuk National University, 52 Naesudong-ro, Heungdeok-gu, Cheongju-si, Chungbuk 28160, Republic of Korea*

[3]*Life Science Research Center, Nine Biopharm Co., LTD. Cheongju-si, Chungbuk, 28161, Republic of Korea*

[4]*Practical Research Division, Honam National Institute of Biological Resources (HNIBR), 99, Gohadoan-gil, Mokpo-si, Jeollanam-do 58762, Republic of Korea*

[*] Co-corresponding Authors: Ji-Won Park, jjiwon87@gmail.com,

Kyung-Seop Ahn, ksahn@kribb.re.kr

Table of Contents

Fig S1.1. UPLC-QTOF-MS data of quercetin........ 2

Fig S1.2. ^{1}H NMR (400 MHz, DMSO-d_6) spectrum of quercetin........ 3

Fig S1.3. ^{13}C NMR (100 MHz, DMSO-d_6) spectrum of quercetin........ 4

Fig S2. Raw data of ExoS-FLAG........ 7

Fig S3.1. Raw data of p-IKKα/β........ 8

Fig S3.2. Raw data of IKKβ........ 9

Fig S3.3. Raw data of pIκBα........ 10

Fig S3.4. Raw data of IκBα........ 11

Fig S3.5. Raw data of p-p65........ 12

Fig S3.6. Raw data of p65........ 13

Fig S3.7. Raw data of β-actin........ 14

Fig S3.8. Raw data of Cytosolic p65........ 15

Fig S3.9. Raw data of Cytosolic β-actin........ 16

Fig S3.10. Raw data of Nuclear p65........ 17

Fig S3.11. Raw data of Nuclear PCNA........ 18

Fig S4.1. Raw data of NLRP3........ 19

Fig S4.2. Raw data of NLRC4........ 20

Fig S4.3. Raw data of Caspase-1........ 21

Fig S4.4. Raw data of β-actin........ 22

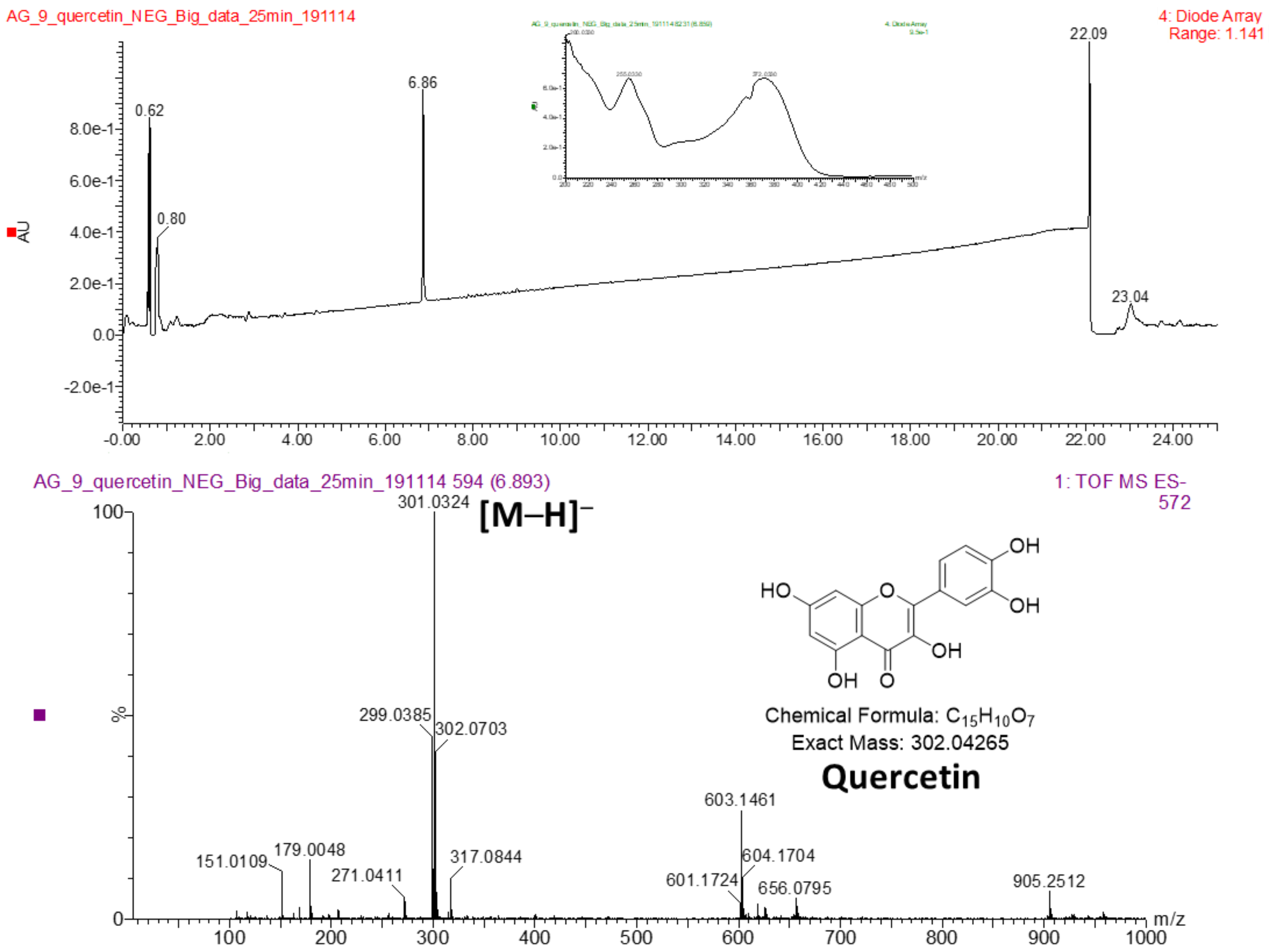

Mass	Calc. Mass	mDa	PPM	DBE	Formula	C	H	O
301.0324	301.0348	-2.4	-8.0	11.5	C15 H9 O7	15	9	7

Fig. S1.1. UPLC-QTOF-MS data of quercetin

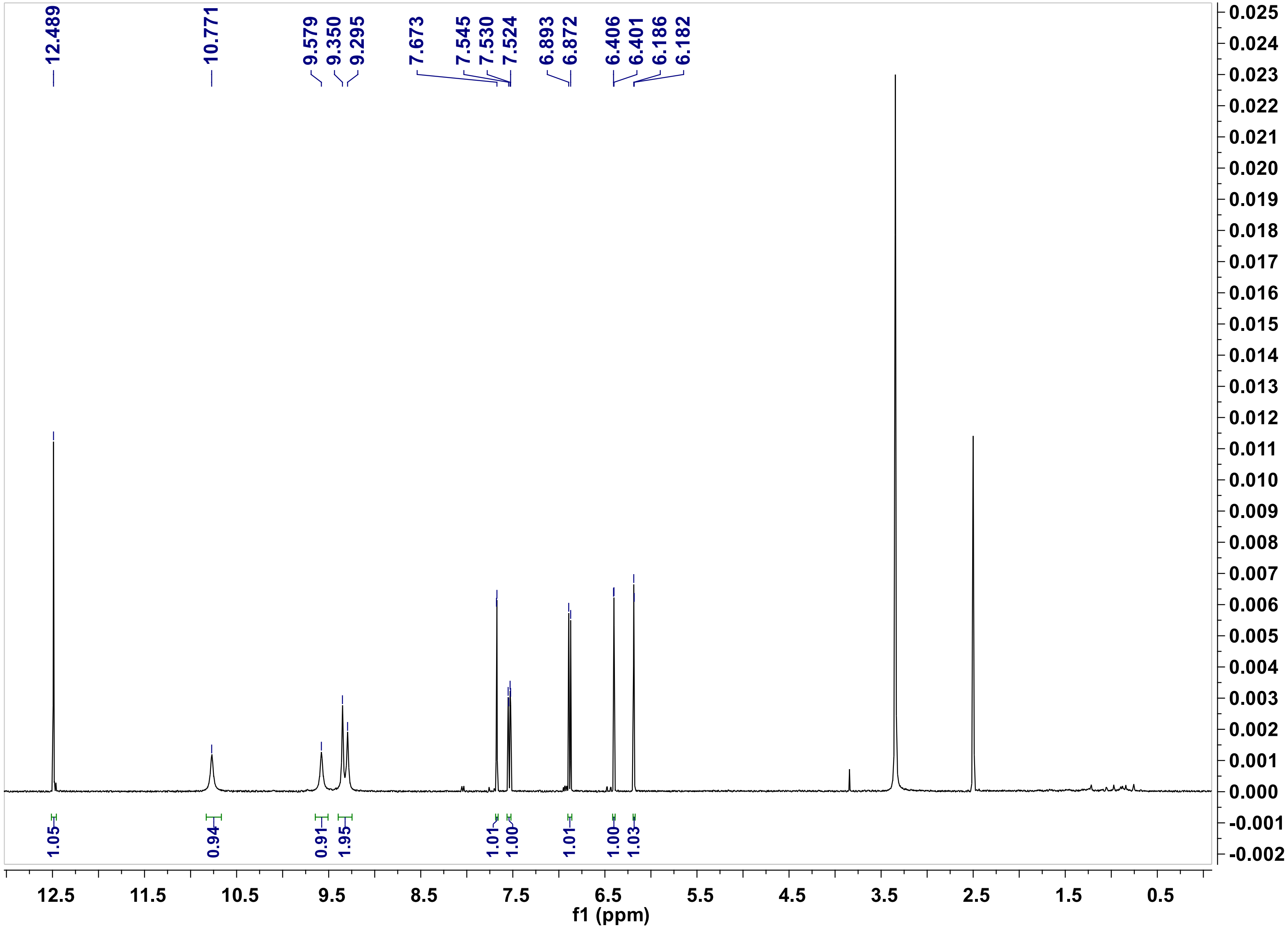

Fig. S1.2. ^{1}H NMR (400 MHz, DMSO-d_6) spectrum of quercetin.

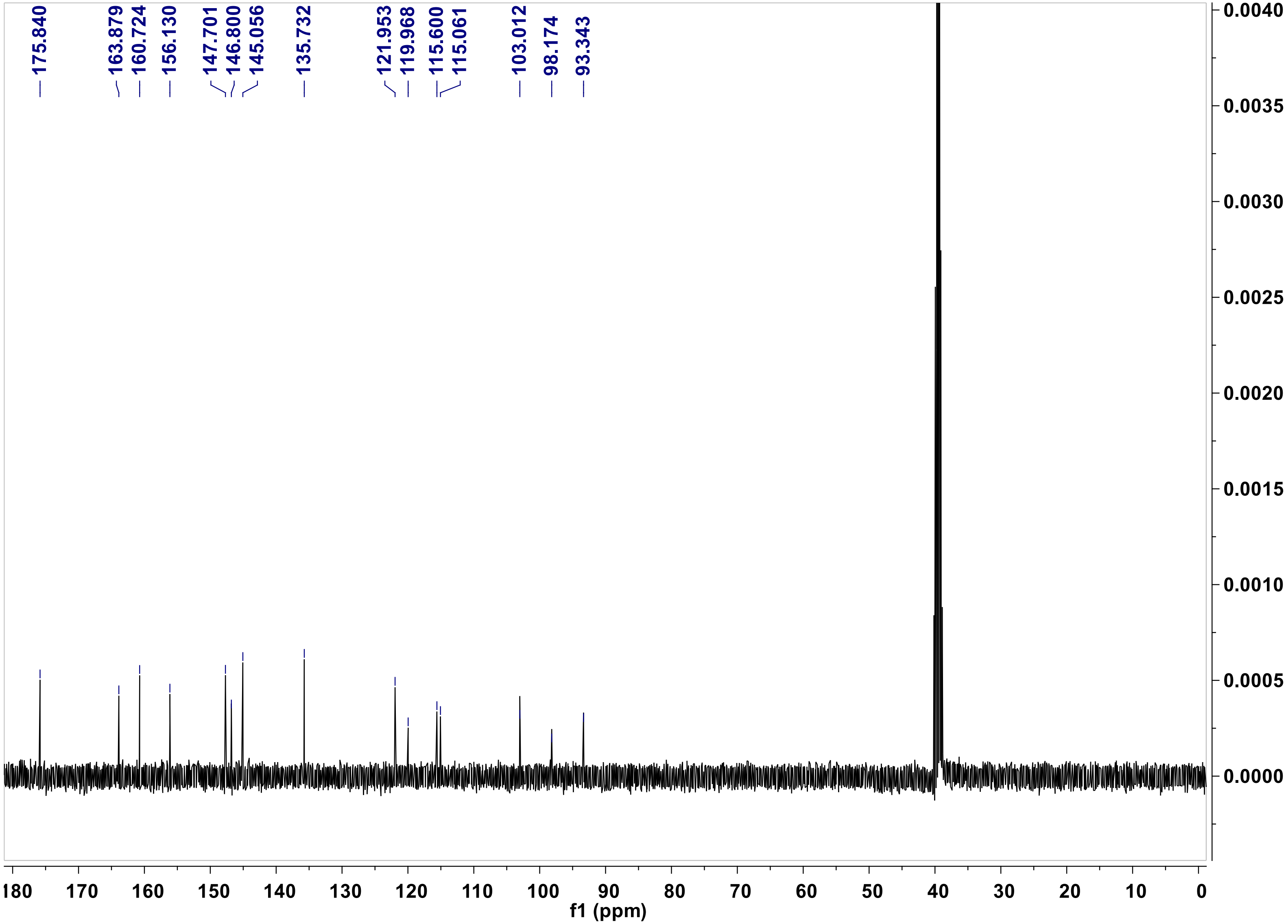

Fig. S1.3. ^{13}C NMR (100 MHz, DMSO-d_6) spectrum of quercetin.

Quercetin

Yellow amorphous powder; ESI-MS *m/z* 301 $[M–H]^-$; ^{1}H NMR (400 MHz, DMSO-d_6) δ_H: 12.49 (1H, s, OH-5), 7.68 (1H, d, J = 2.0 Hz, H-2′), 7.54 (1H, d, J = 8.4, 2.0 Hz, H-6′), 6.88 (1H, d, J = 8.4 Hz, H-5′), 6.40 (1H, d, J = 2.0 Hz, H-8) 6.18 (1H, d, J = 2.0 Hz, H-6); ^{13}C-NMR (100 MHz, DMSO-d_6) δ_C: 175.8 (C-4), 163.9 (C-7), 160.7 (C-5), 156.1 (C-9), 147.7 (C-2), 146.8 (C-4′), 145.1 (C-3′), 135.7 (C-3), 122.0 (C-1′), 120.0 (C-6′), 115.6 (C-5′), 115.1 (C-2′), 103.0 (C-10), 98.2 (C-6), 93.3 (C-8).

References

Xu, J., Li, X., Zhang, P., Li, Z. L., Wang, Y., 2005. Antiinflammatory constituents from the roots of *Smilax bockii* warb. *Arch. Pharm. Res.* 28, 395–399.

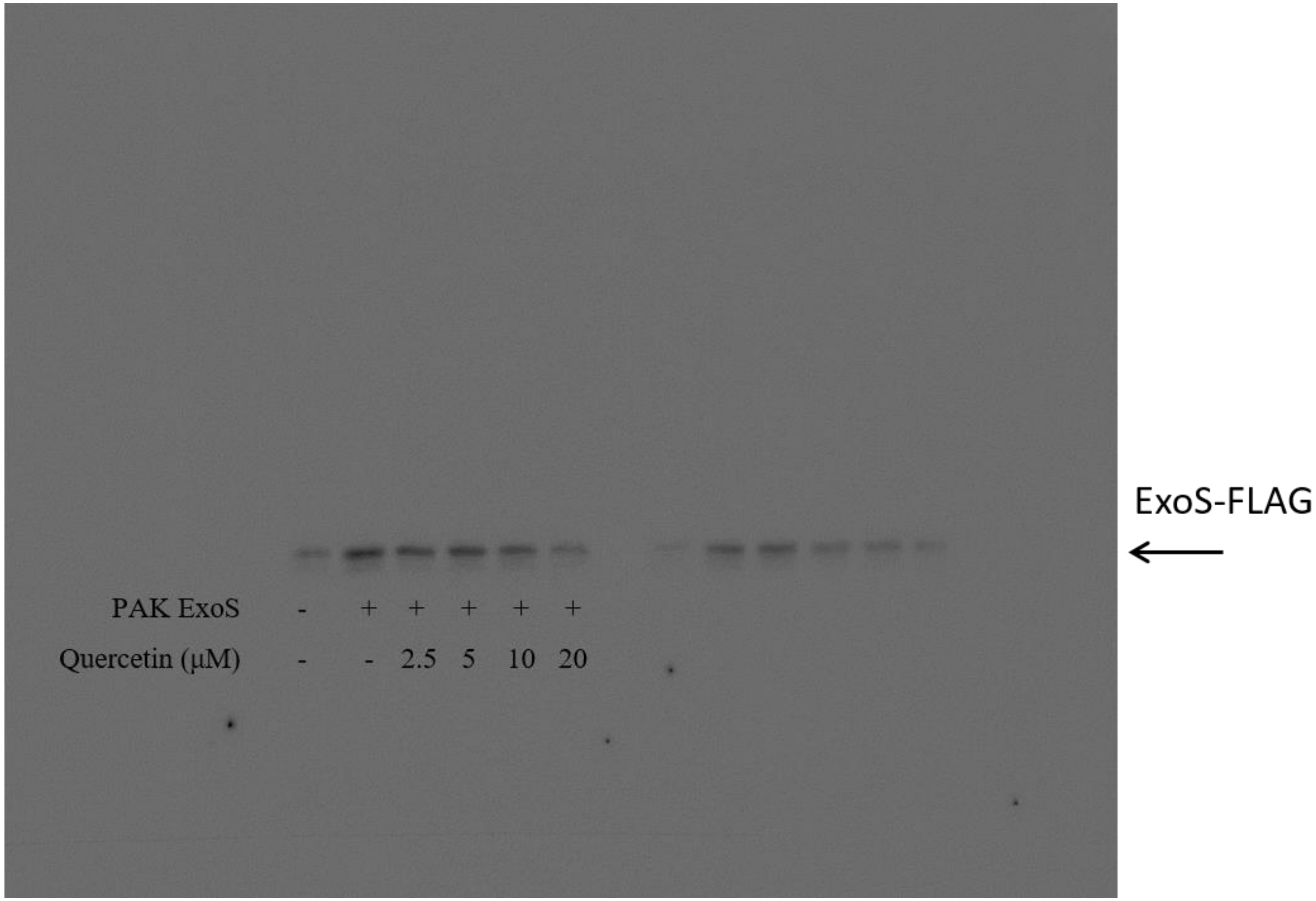

Fig S2. Raw data of ExoS-FLAG

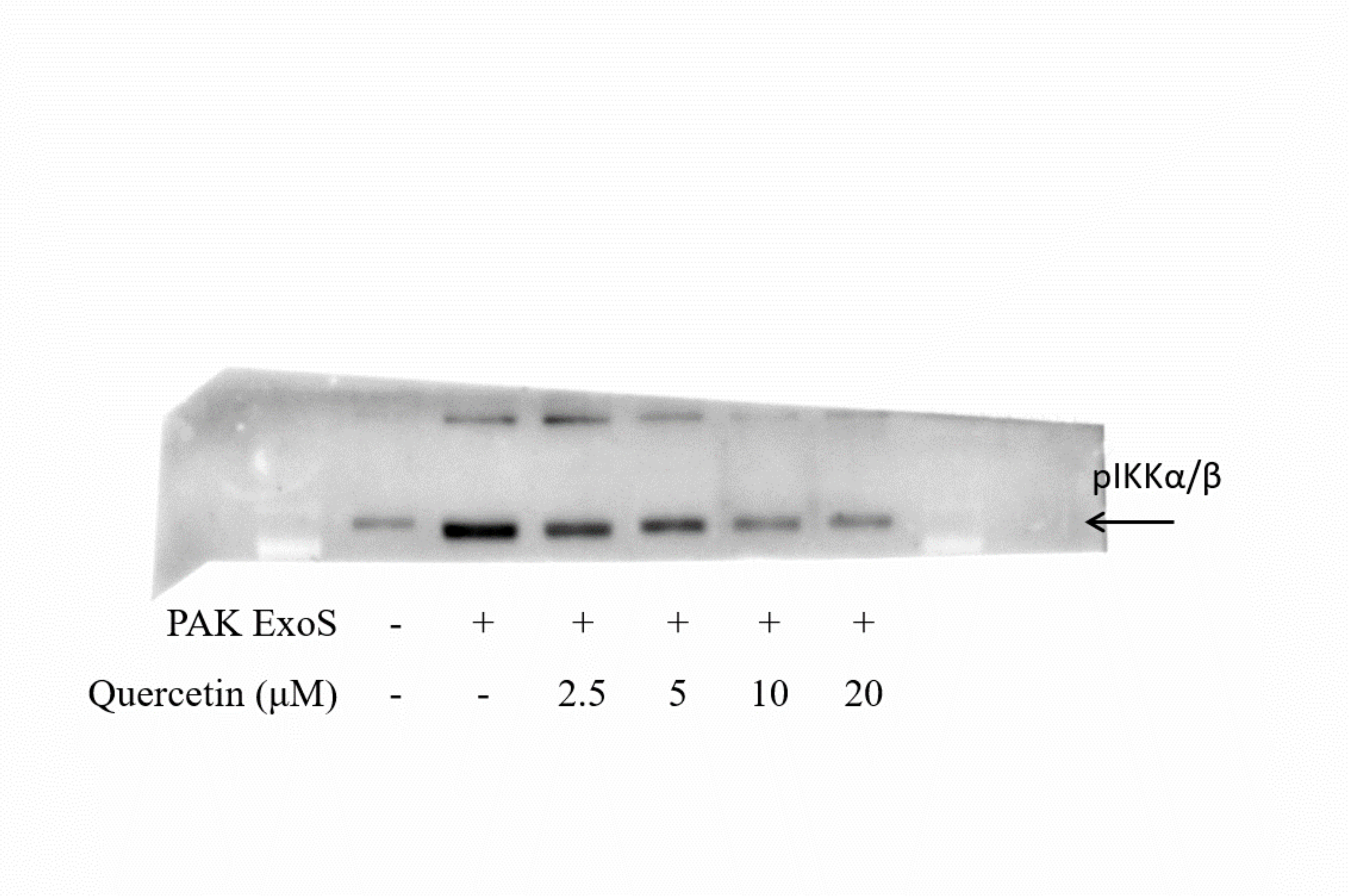

Fig S3.1. Raw data of p-IKKα/β

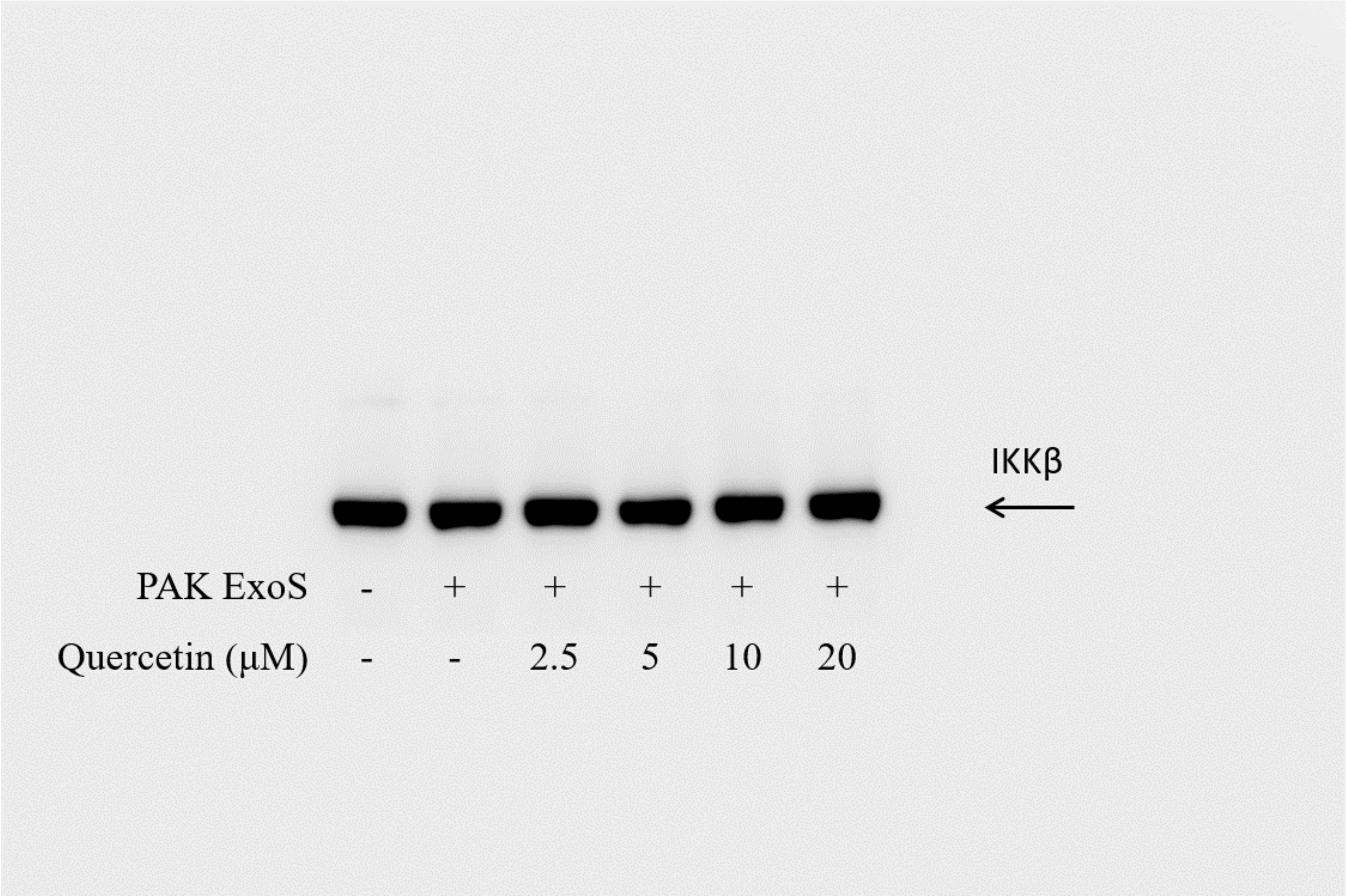

Fig S3.2. Raw data of p-IKKβ

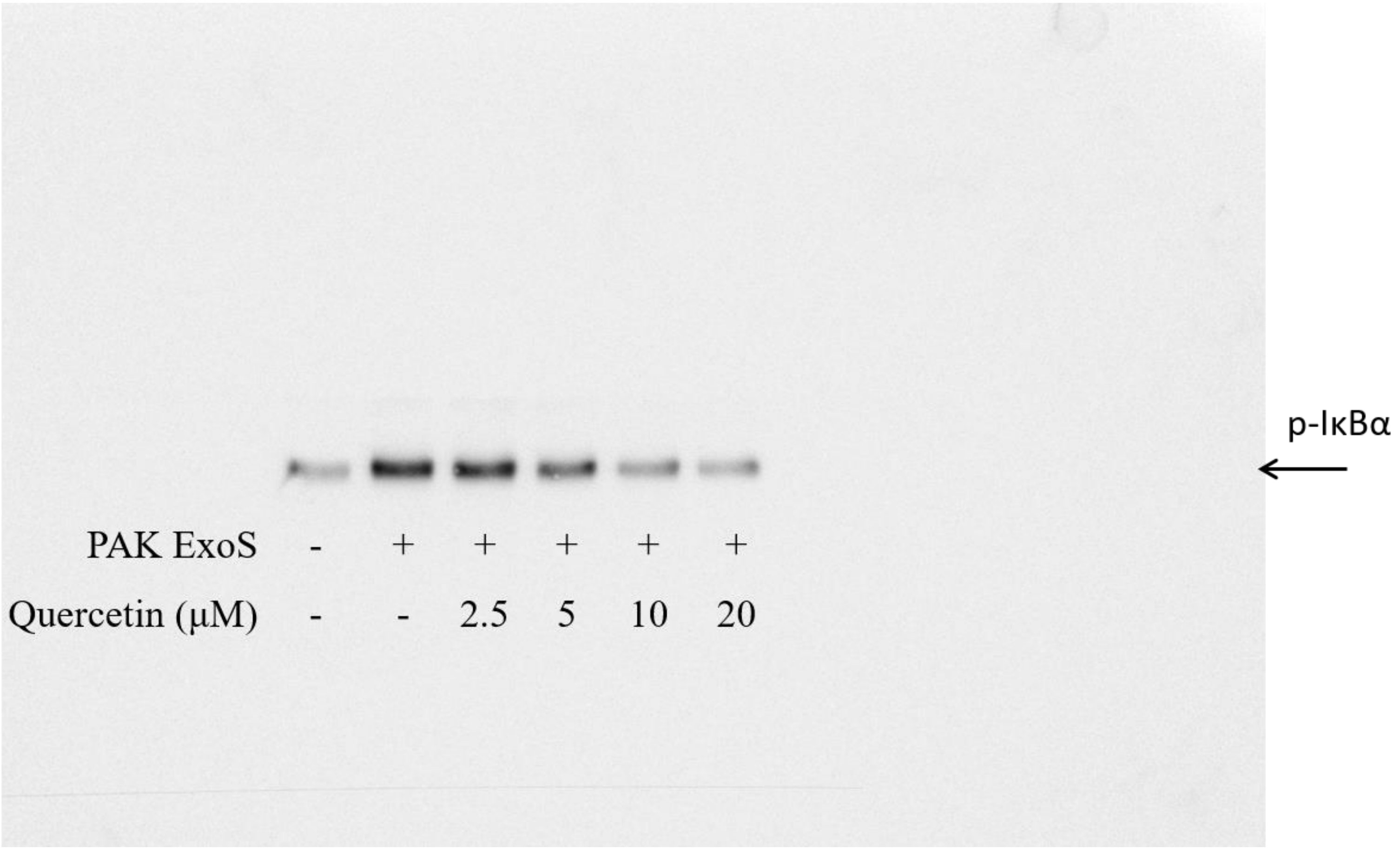

Fig S3.3. Raw data of p-IκBα

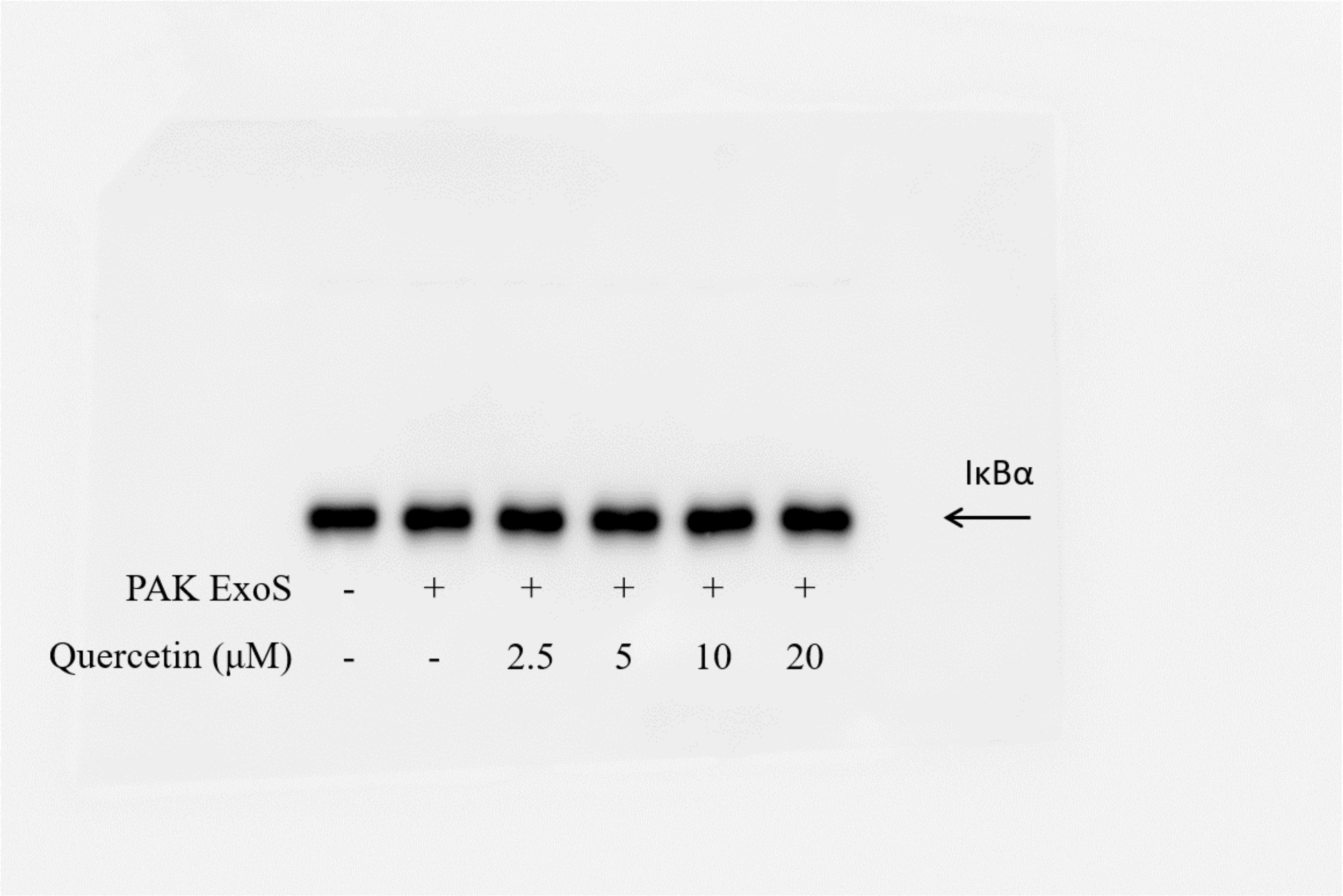

Fig S3.4. Raw data of IκBα

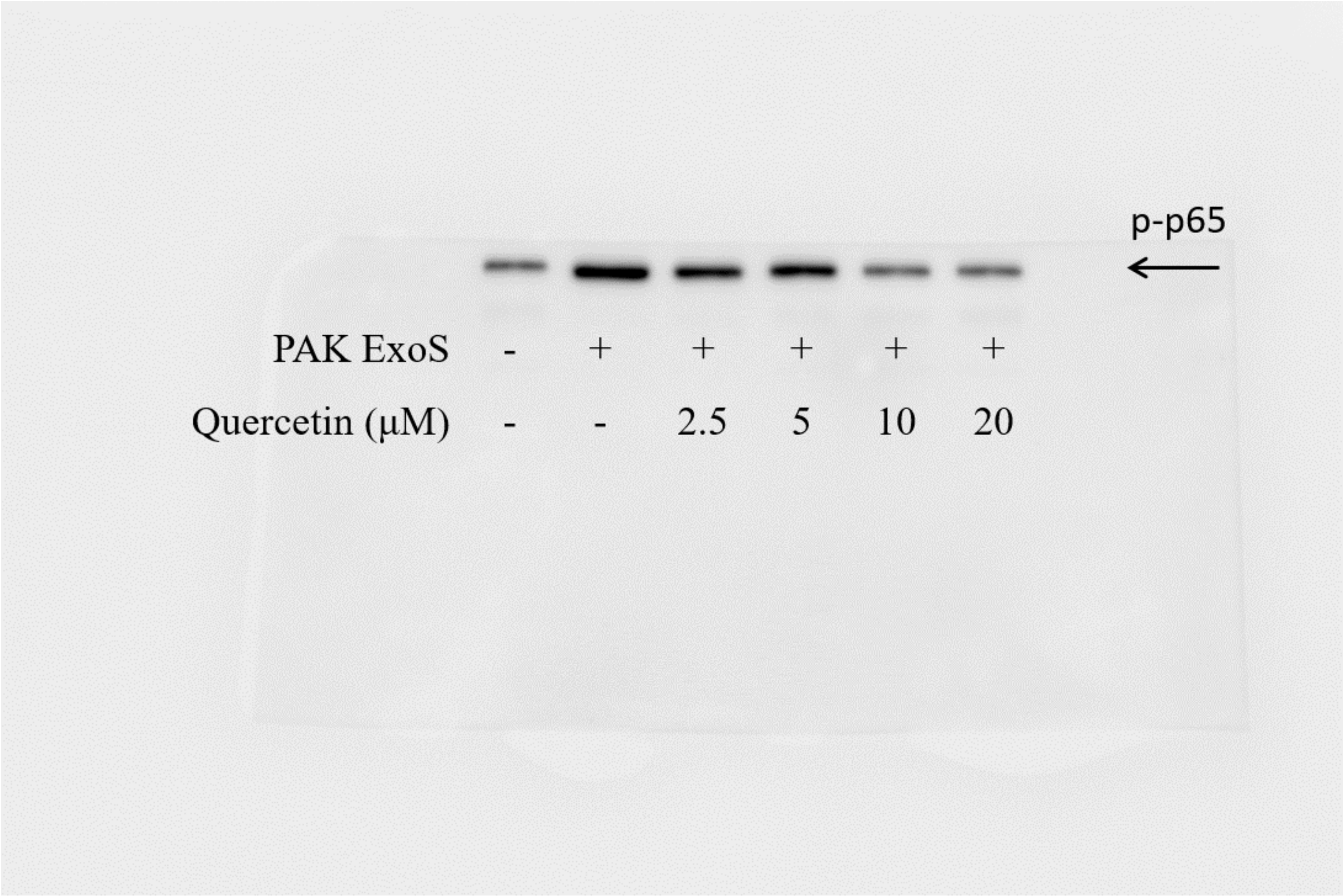

Fig S3.5. Raw data of p-p65

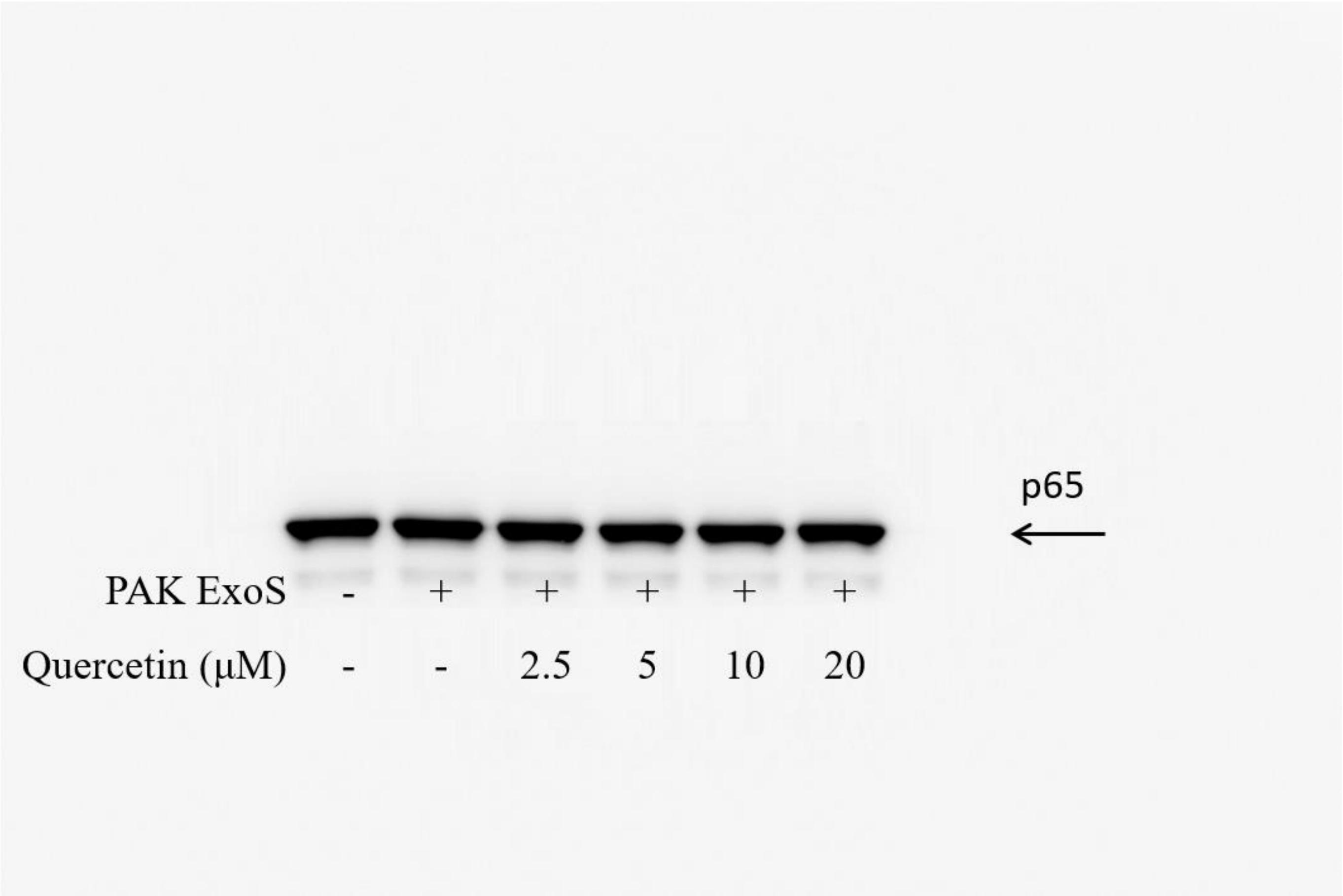

Fig S3.6. Raw data of p65

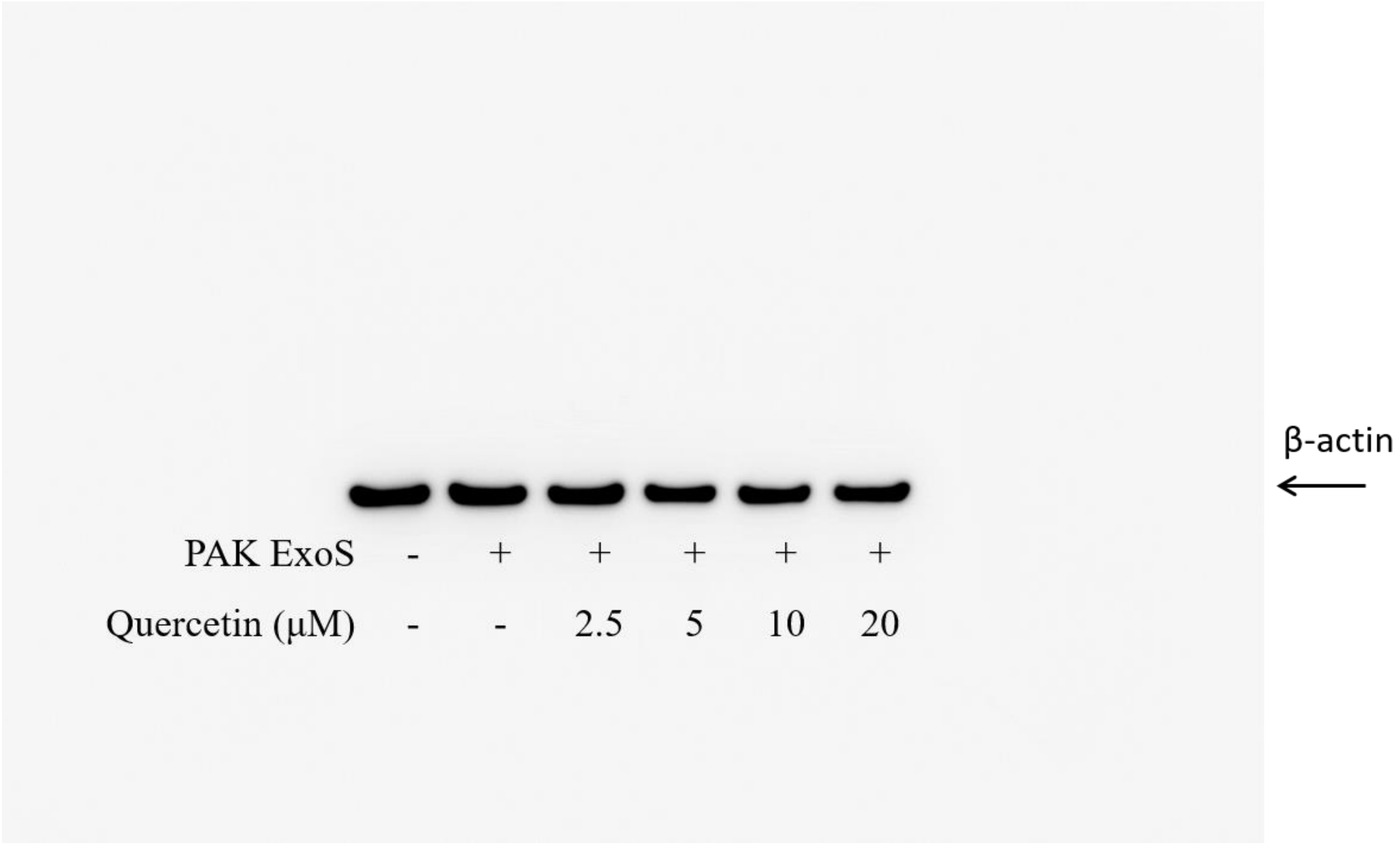

Fig S3.7. Raw data of β-actin

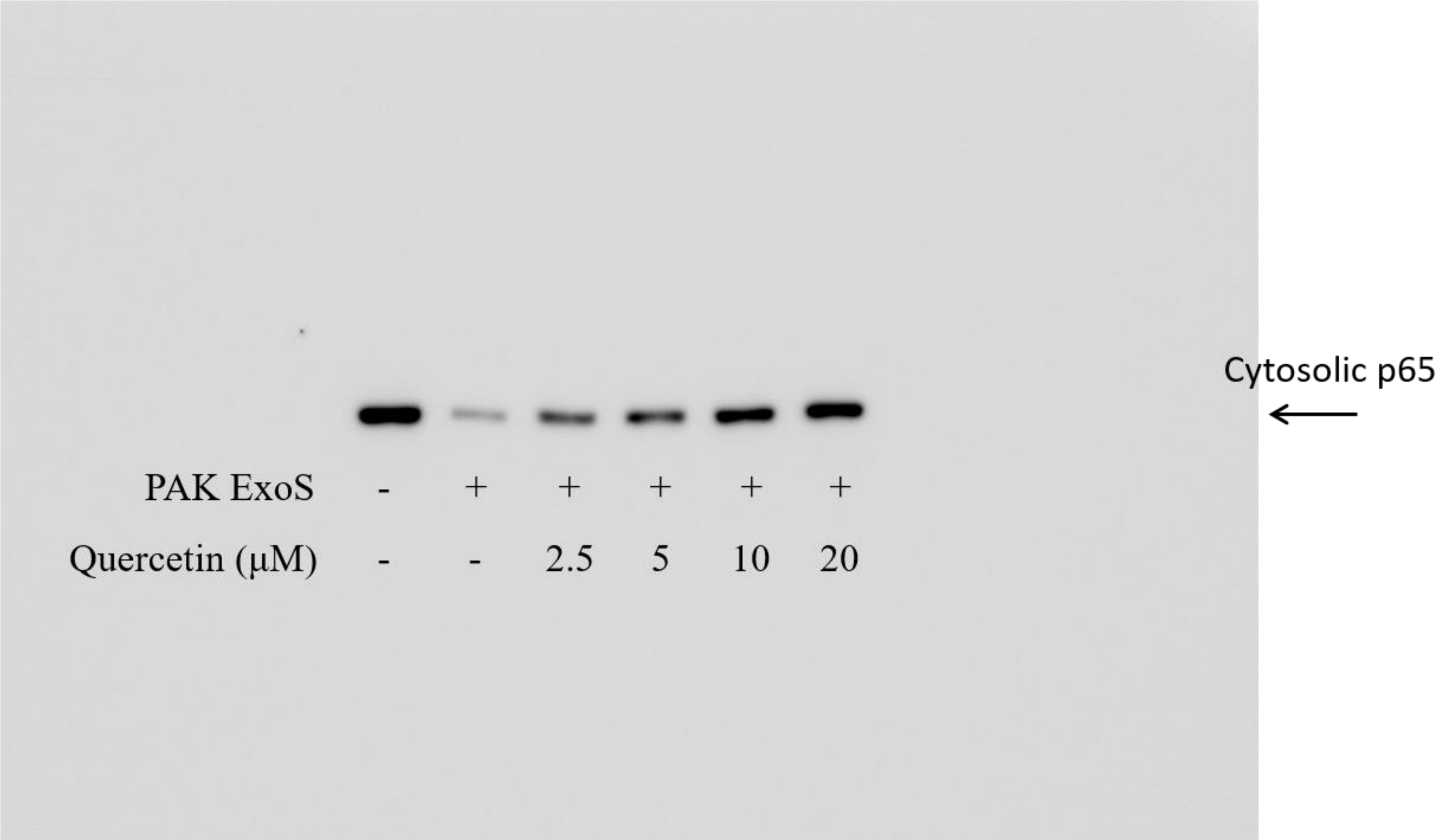

Fig S3.8. Raw data of Cytosolic p65

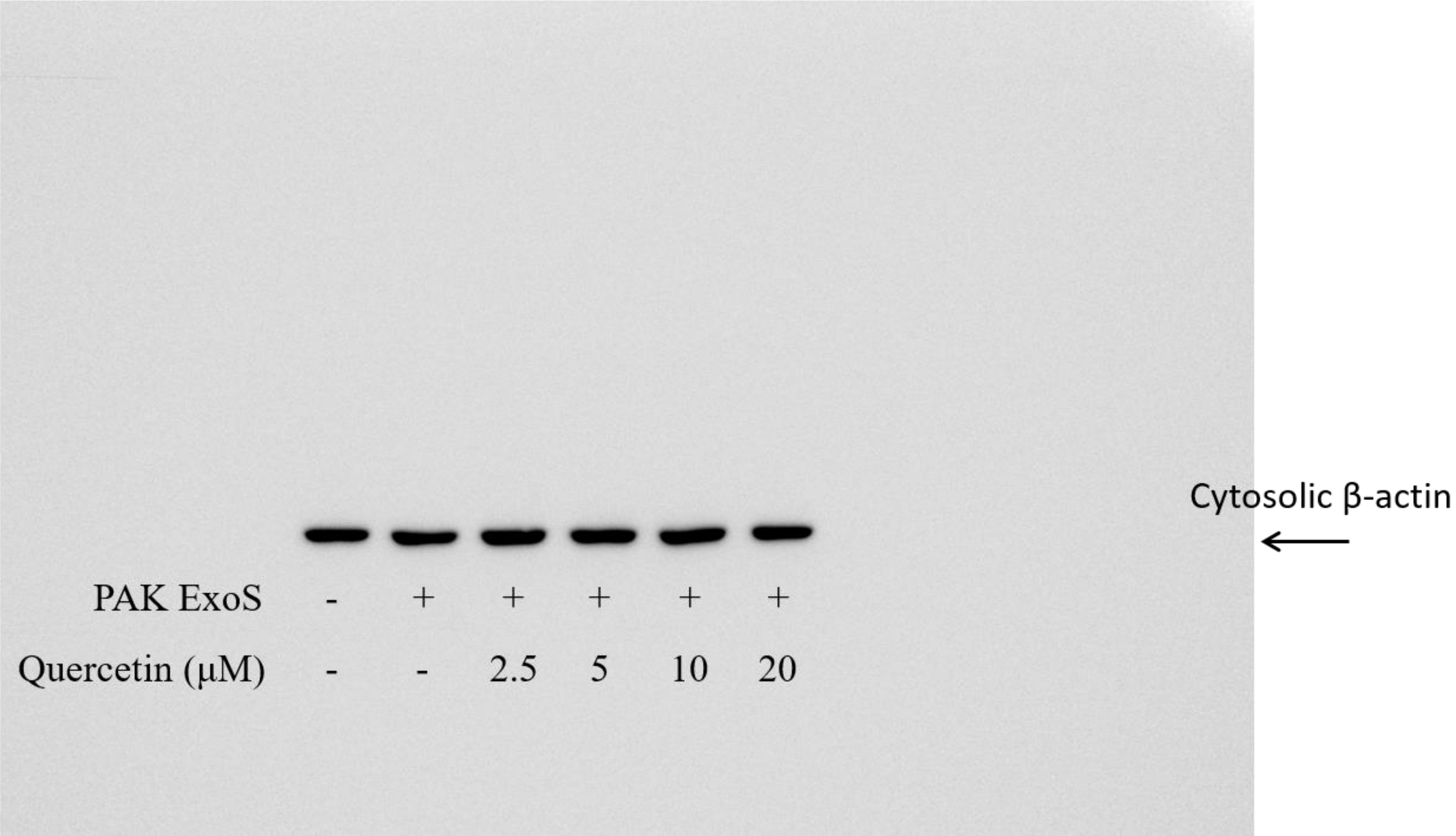

Fig S3.9. Raw data of Cytosolic β-actin

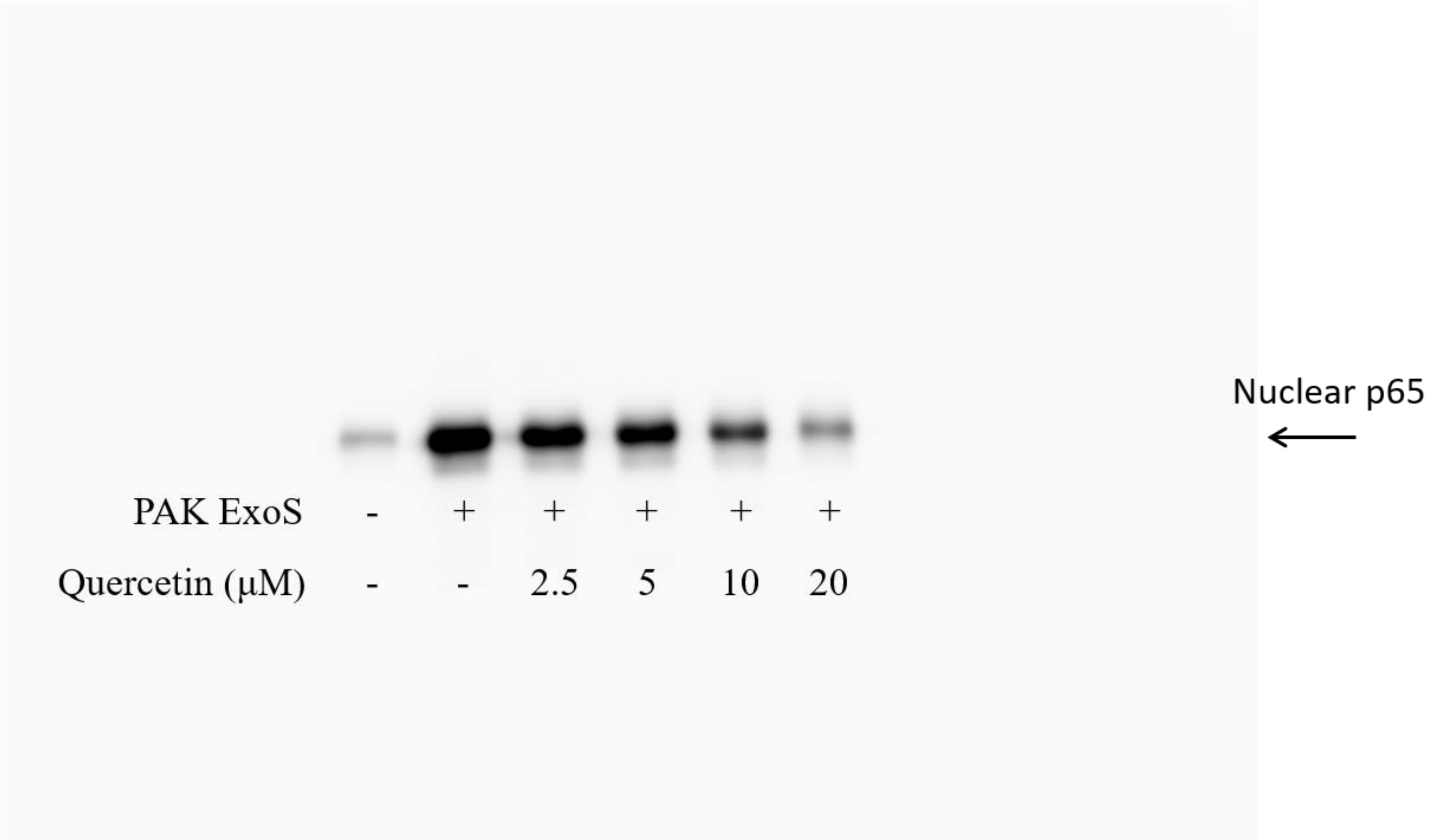

Fig S3.10. Raw data of Nuclear p65

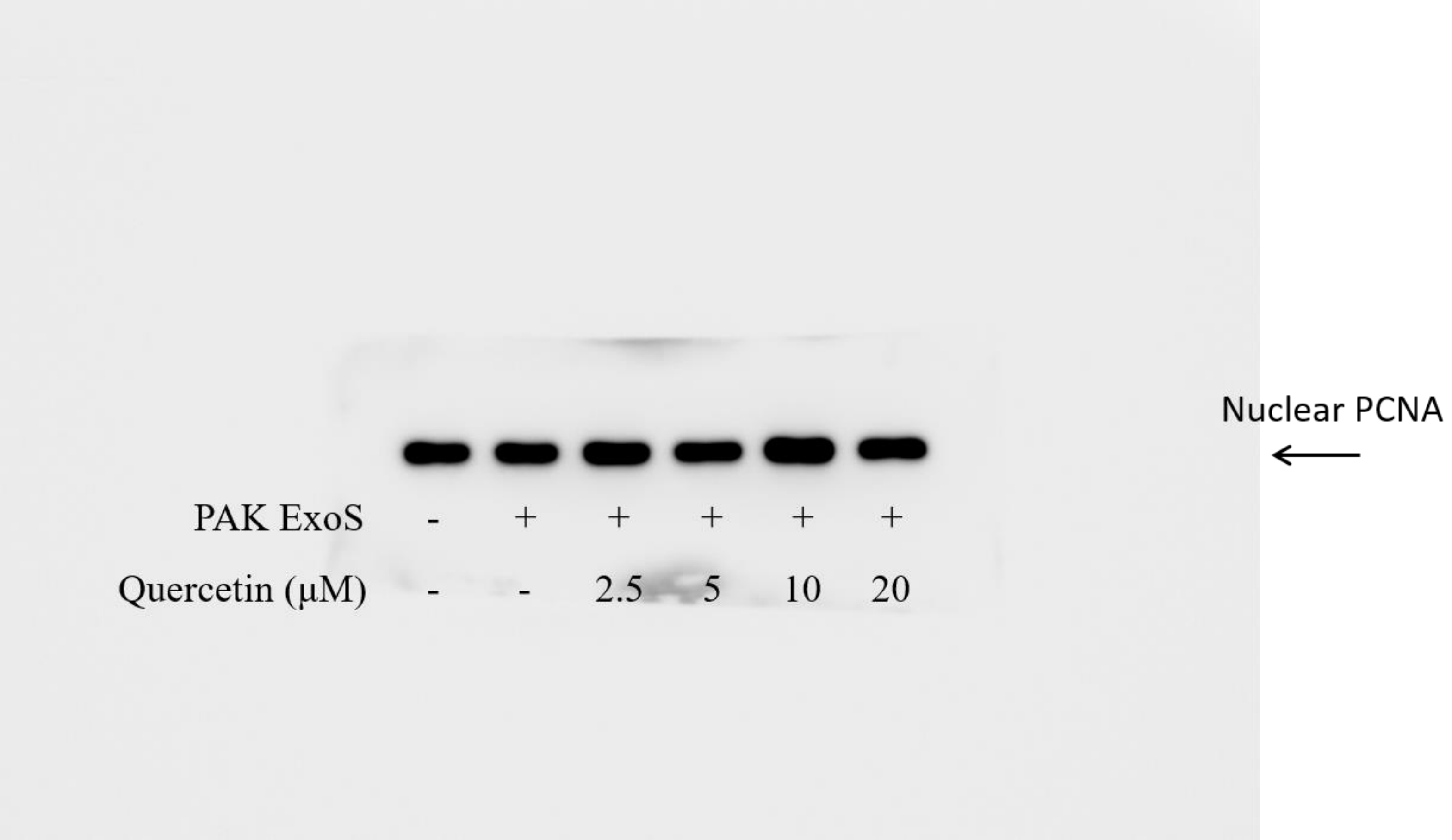

Fig S3.11. Raw data of Nuclear PCNA

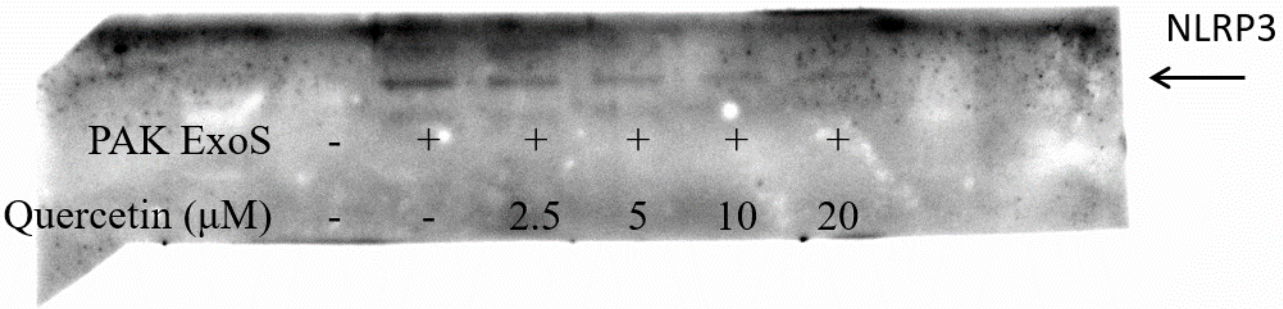

Fig S4.1. Raw data of NLRP3

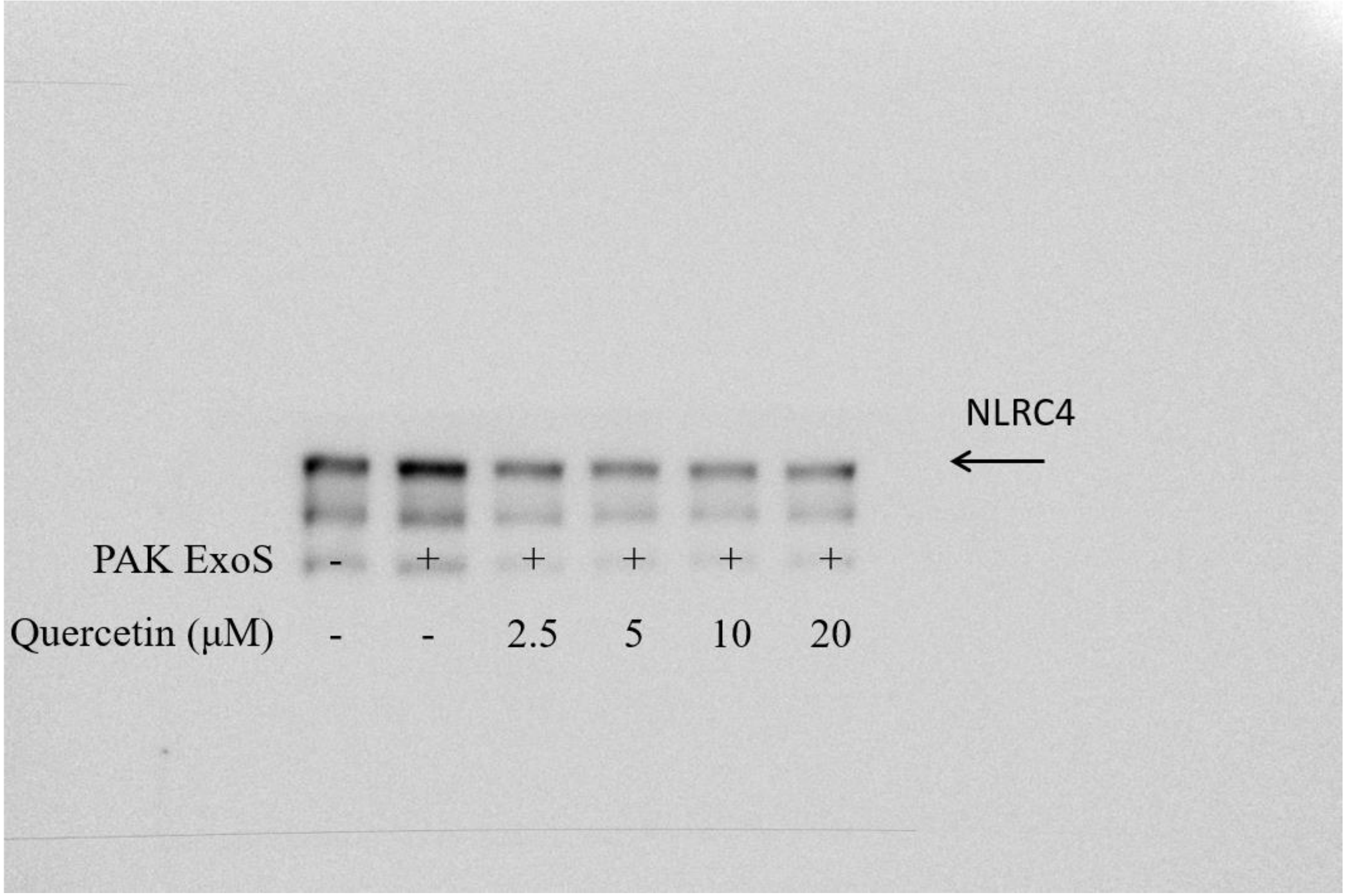

Fig S4.2. Raw data of NLRC4

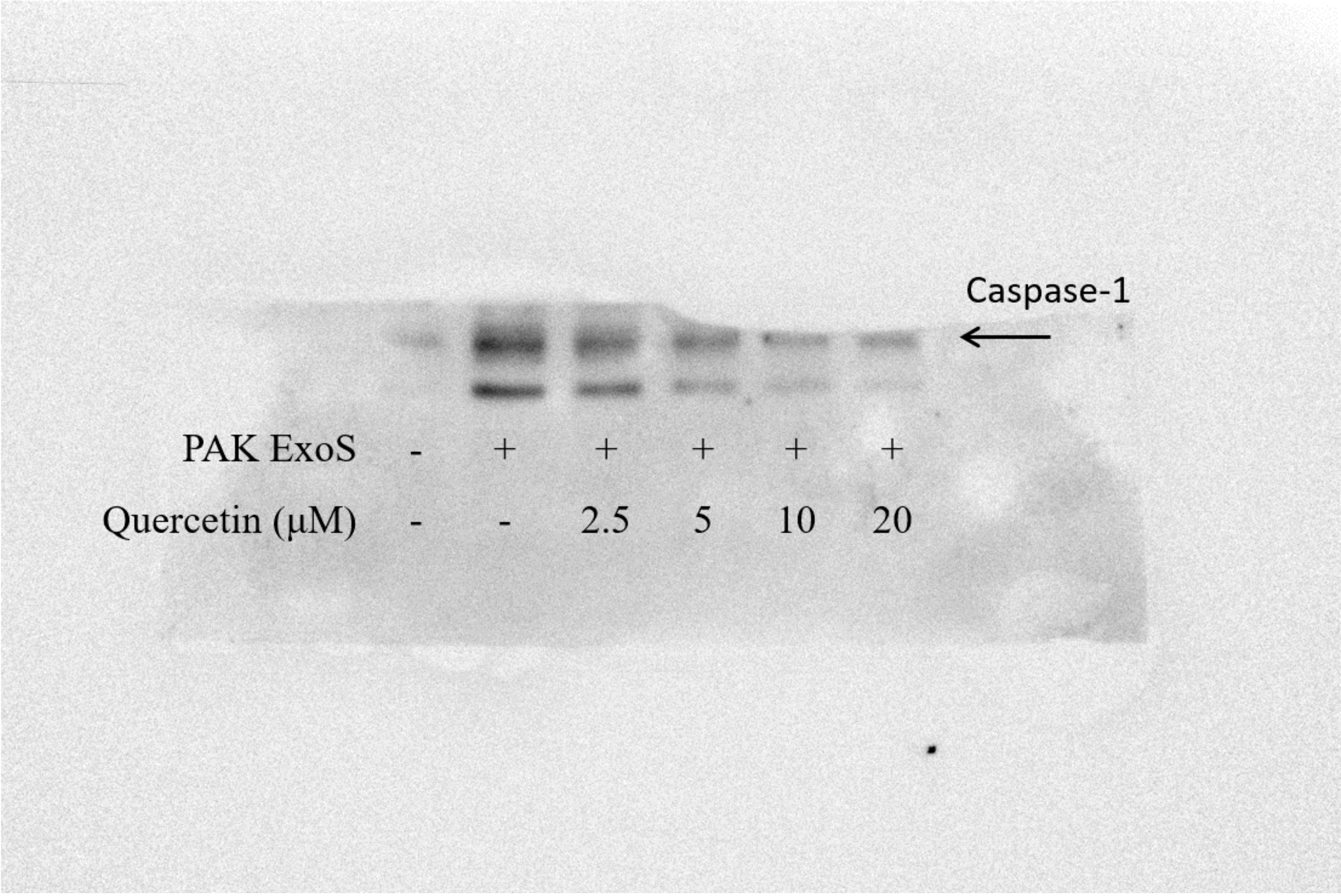

Fig S4.3. Raw data of Caspase-I

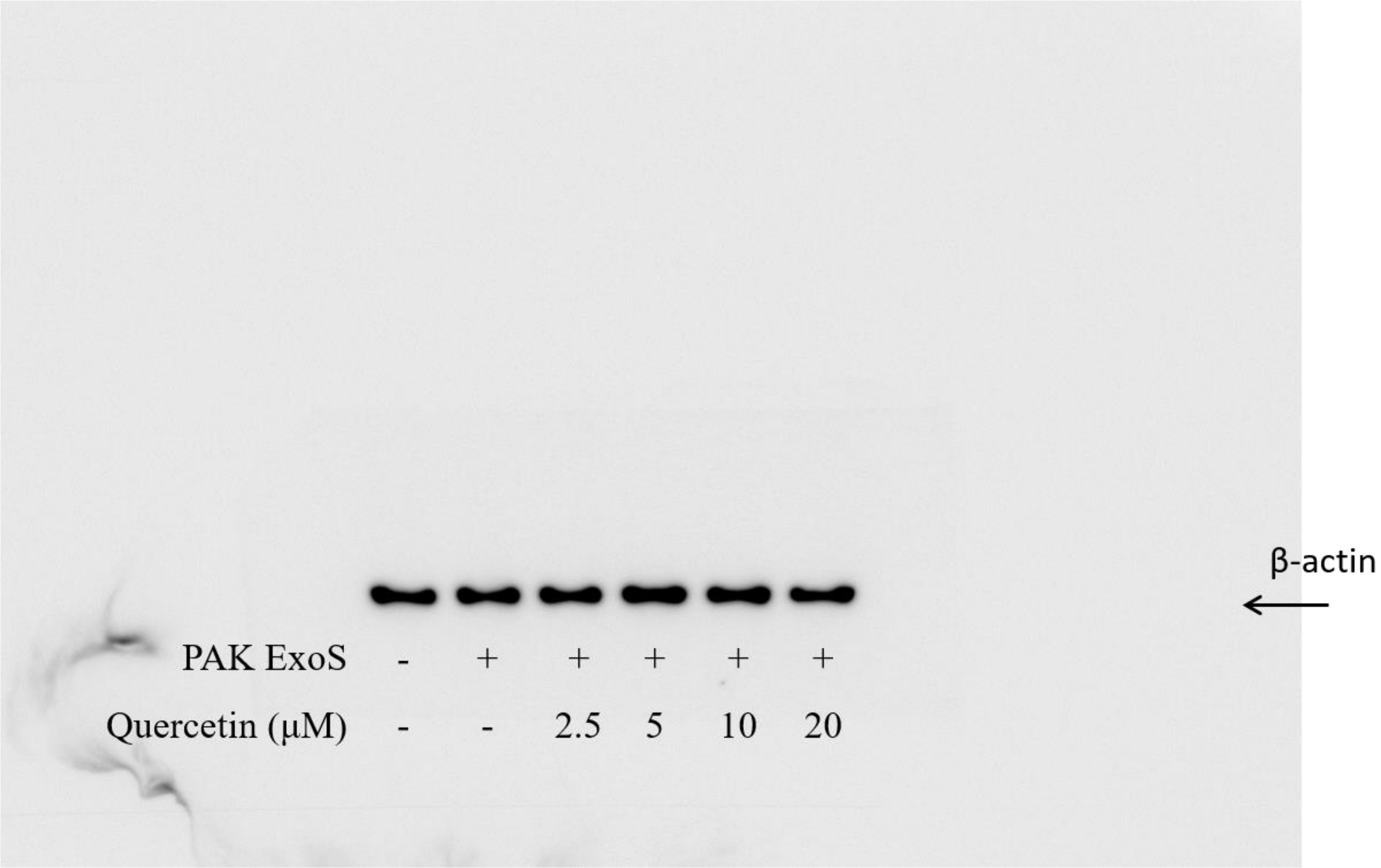

Fig S4.4. Raw data of β-actin
